# Supplementary material for: Hyperconnectivity of two separate long-range cholinergic systems contributes to the reorganization of the brain functional connectivity during nicotine withdrawal in male mice
Source: bioRxiv. 2023 Mar 31:2023.03.29.534836. Preprint. [Version 1] doi: 10.1101/2023.03.29.534836 (PMC10081261; doi:10.1101/2023.03.29.534836)
Supplement: Supplement 1 [file media-1.pdf]

Extended DATA for: **Hyperconnectivity of two separate long-range cholinergic systems contributes to the reorganization of the brain functional connectivity during nicotine withdrawal in male mice**

Lieselot L.G. Carrette[1]*, Adam Kimbrough[1#], Pasha A. Davoudian[2,3], Alex C. Kwan[4], Andres Collazo[5], Olivier George[1]*

[1] Department of Psychiatry, UC San Diego, La Jolla, CA, 92032, United States
[2] Medical Scientist Training Program, Yale University School of Medicine, New Haven, CT, 06511, United States
[3] Interdepartmental Neuroscience Program, Yale University School of Medicine, New Haven, CT, 06511, United States
[4] Meinig School of Biomedical Engineering, Cornell University, Ithaca, NY, 14853, United States
[5] Beckman Institute, CalTech, Pasadena, CA, 91125, United States

*Correspondence to: lcarrette@health.ucsd.edu or olgeorge@health.ucsd.edu

saline

Region	Module
Posterior limiting nucleus of the thalamus	13
Endopiriform nucleus	
Olfactory tubercle	
Central amygdalar nucleus	
Basolateral amygdalar nucleus	
Globus pallidus external segment	12
Posterior complex of the thalamus	
Ventral anterior–lateral complex of the thalamus	
Piriform–amygdalar area	11
Diagonal band nucleus	
Ventrolateral preoptic nucleus	
Cortical amygdalar area posterior part	
Anteroventral preoptic nucleus	
Nucleus of the lateral olfactory tract	
Bed nucleus of the accessory olfactory tract	
Parataenial nucleus	
Interanteromedial nucleus of the thalamus	
Magnocellular nucleus	10
Globus pallidus internal segment	
Temporal association areas	
Ventral auditory area	
Posterior auditory area	
Lateral amygdalar nucleus	
Dorsal part of the lateral geniculate complex	
Primary auditory area	
Postpiriform transition area	
Mammillary body	
Ventral posterolateral nucleus of the thalamus	
Field CA2	
Dorsal auditory area	
Entorhinal area lateral part	
Ansiform lobule	
Entorhinal area medial part	
Intergeniculate leaflet of the lateral geniculate complex	
Ectorhinal area	9
Perirhinal area	
Reticular nucleus of the thalamus	
Induseum griseum	
Interpeduncular nucleus	
Ventral posterior complex of the thalamus	
Red nucleus	
Preparasubthalamic nucleus	
Nucleus of the brachium of the inferior colliculus	
Medial geniculate complex	8
Ventral tegmental area	
Substantia innominata	
Medial amygdalar nucleus	
Pons	
Retrochiasmatic area	
Field CA3	
Ventral part of the lateral geniculate complex	
Substantia nigra reticular part	
Zona incerta	
Paraventricular nucleus of the thalamus	7
Medial habenula	
Frontal pole cerebral cortex	
Supplemental somatosensory area	
Anterior olfactory nucleus	
Central medial nucleus of the thalamus	
Intercalated amygdalar nucleus	
Medial preoptic area	
Intermediodorsal nucleus of the thalamus	
Visceral area	6
Caudoputamen	
Orbital area ventrolateral part	
Anterior amygdalar area	
Primary somatosensory area nose	5
Primary somatosensory area mouth	
Claustrum	
Anteroventral nucleus of thalamus	
Supraoptic nucleus	
Gustatory areas	
Taenia tecta	
Prelimbic area	
Piriform area	
Agranular insular area ventral part	
Dorsal peduncular area	
Primary somatosensory area upper limb	
Supramammillary nucleus	
Anterior cingulate area dorsal part	
Orbital area medial part	
Infralimbic area	
Median preoptic nucleus	
Lateral preoptic area	
Periventricular hypothalamic nucleus preoptic part	

Region	Module
Paraventricular hypothalamic nucleus descending division	4
Medial pretectal area	
Anterior group of the dorsal thalamus	
Dorsomedial nucleus of the hypothalamus	
Nucleus accumbens	
Triangular nucleus of septum	3
Lateral hypothalamic area	
Tuberal nucleus	
Posterior amygdalar nucleus	
Periventricular hypothalamic nucleus posterior part	
Ventral medial nucleus of the thalamus	
Subthalamic nucleus	
Arcuate hypothalamic nucleus	
Ventromedial hypothalamic nucleus	
Posterior hypothalamic nucleus	
Pedunculopontine nucleus	
Culmen	
Paracentral nucleus	
Substantia nigra compact part	
Anterior pretectal nucleus	
Nucleus of Darkschewitsch	
Field CA1	
Nucleus of the posterior commissure	
Submedial nucleus of the thalamus	
Subparafascicular nucleus	
Simple lobule	
Paraflocculus	
Thalamus sensory–motor cortex related	
Subiculum	
Midbrain reticular nucleus	
Central lobule	
Parafascicular nucleus	
Peripeduncular nucleus	
Nucleus of reuniens	
Posterior pretectal nucleus	
Lateral habenula	
Nucleus of the optic tract	2
Postsubiculum	
Parasubiculum	
Lateral posterior nucleus of the thalamus	
Midbrain reticular nucleus retrorubral area	
Presubiculum	
Dentate gyrus	
Fundus of striatum	
Cuneiform nucleus	
Inferior colliculus	
Superior colliculus motor related	
Vestibular nuclei	
Superior colliculus sensory related	
Parasubthalamic nucleus	
Primary visual area	
Pons motor related	
Precommissural nucleus	
Retrosplenial area ventral part	
Interanterodorsal nucleus of the thalamus	
Anterior hypothalamic nucleus	
Posterolateral visual area	
Primary somatosensory area barrel field	1
Agranular insular area posterior part	
Anteromedial visual area	
Lateral dorsal nucleus of thalamus	
Primary somatosensory area trunk	
Lateral septal complex	
Primary somatosensory area lower limb	
Primary motor area	
Medial preoptic nucleus	
Anterodorsal preoptic nucleus	
Fasciola cinerea	
Anterodorsal nucleus	
Periaqueductal gray	
Parastrial nucleus	
Pontine reticular nucleus	
Lateral visual area	
Anterolateral visual area	
Retrosplenial area lateral agranular part	
Bed nuclei of the stria terminalis	
Anteroventral periventricular nucleus	
Posteromedial visual area	
Retrosplenial area dorsal part	
Mediodorsal nucleus of thalamus	
Orbital area lateral part	
Paraventricular hypothalamic nucleus	
Septofimbrial nucleus	
Periventricular zone	
Secondary motor area	
Olivary pretectal nucleus	
Central lateral nucleus of the thalamus	
Medial septal nucleus	
Subparaventricular zone	
Anterior cingulate area ventral part	

nicotine

Region	Module
Bed nucleus of the accessory olfactory tract	9
Frontal pole cerebral cortex	
Ventral posterior complex of the thalamus	
Medial amygdalar nucleus	
Bed nuclei of the stria terminalis	
Diagonal band nucleus	
Substantia innominata	
Anterior olfactory nucleus	
Medial preoptic nucleus	
Primary somatosensory area barrel field	
Anterior hypothalamic nucleus	
Posterior complex of the thalamus	
Ventral anterior–lateral complex of the thalamus	
Orbital area lateral part	
Lateral dorsal nucleus of thalamus	
Orbital area ventrolateral part	
Lateral posterior nucleus of the thalamus	8
Nucleus of the lateral olfactory tract	
Anterior group of the dorsal thalamus	
Primary somatosensory area mouth	
Supraoptic nucleus	7
Dorsal part of the lateral geniculate complex	
Olfactory tubercle	
Orbital area medial part	
Globus pallidus external segment	
Preparasubthalamic nucleus	6
Primary somatosensory area nose	
Taenia tecta	
Anterior amygdalar area	
Subparaventricular zone	
Secondary motor area	
Submedial nucleus of the thalamus	
Primary somatosensory area trunk	
Substantia nigra reticular part	
Paracentral nucleus	
Subthalamic nucleus	
Entorhinal area medial part	
Parasubiculum	
Cuneiform nucleus	
Posterior amygdalar nucleus	
Tuberal nucleus	
Median preoptic nucleus	
Reticular nucleus of the thalamus	
Periventricular hypothalamic nucleus preoptic part	
Anteroventral preoptic nucleus	
Field CA1	
Field CA2	
Agranular insular area ventral part	
Retrochiasmatic area	
Infralimbic area	
Anteroventral periventricular nucleus	
Lateral hypothalamic area	
Ventral medial nucleus of the thalamus	
Globus pallidus internal segment	
Magnocellular nucleus	
Nucleus accumbens	
Medial preoptic area	
Lateral preoptic area	
Primary somatosensory area upper limb	
Dorsal peduncular area	
Primary motor area	
Zona incerta	
Field CA3	
Ventromedial hypothalamic nucleus	
Anterodorsal preoptic nucleus	
Primary somatosensory area lower limb	
Parastrial nucleus	
Primary visual area	
Interanterodorsal nucleus of the thalamus	5
Paraventricular nucleus of the thalamus	4
Parataenial nucleus	
Induseum griseum	
Triangular nucleus of septum	
Lateral habenula	
Intergeniculate leaflet of the lateral geniculate complex	
Precommissural nucleus	
Nucleus of the posterior commissure	
Olivary pretectal nucleus	
Nucleus of the optic tract	
Medial habenula	
Medial pretectal area	
Interanteromedial nucleus of the thalamus	
Thalamus sensory–motor cortex related	
Septofimbrial nucleus	

Region	Module
Medial septal nucleus	3
Dentate gyrus	
Posterolateral visual area	
Anteromedial visual area	
Substantia nigra compact part	
Paraventricular hypothalamic nucleus descending division	
Nucleus of reuniens	
Dorsomedial nucleus of the hypothalamus	
Subiculum	
Parasubthalamic nucleus	
Fundus of striatum	
Caudoputamen	
Arcuate hypothalamic nucleus	
Posterior limiting nucleus of the thalamus	
Periventricular zone	
Paraflocculus	
Anterior cingulate area dorsal part	
Paraventricular hypothalamic nucleus	
Supplemental somatosensory area	
Periventricular hypothalamic nucleus posterior part	
Ansiform lobule	
Presubiculum	
Posteromedial visual area	
Intercalated amygdalar nucleus	
Central amygdalar nucleus	
Mammillary body	
Gustatory areas	
Postsubiculum	
Anterolateral visual area	
Supramammillary nucleus	
Lateral visual area	
Anterodorsal nucleus	2
Posterior pretectal nucleus	
Nucleus of Darkschewitsch	
Central lateral nucleus of the thalamus	
Anterior pretectal nucleus	
Parafascicular nucleus	
Ventral posterolateral nucleus of the thalamus	1
Piriform–amygdalar area	
Ventrolateral preoptic nucleus	
Lateral septal complex	
Postpiriform transition area	
Dorsal auditory area	
Entorhinal area lateral part	
Endopiriform nucleus	
Cortical amygdalar area posterior part	
Central medial nucleus of the thalamus	
Prelimbic area	
Intermediodorsal nucleus of the thalamus	
Agranular insular area posterior part	
Piriform area	
Ectorhinal area	
Perirhinal area	
Primary auditory area	
Ventral auditory area	
Visceral area	
Posterior auditory area	
Temporal association areas	
Anteroventral nucleus of thalamus	
Vestibular nuclei	
Peripeduncular nucleus	
Simple lobule	
Fasciola cinerea	
Inferior colliculus	
Periaqueductal gray	
Culmen	
Ventral part of the lateral geniculate complex	
Mediodorsal nucleus of thalamus	
Nucleus of the brachium of the inferior colliculus	
Retrosplenial area ventral part	
Lateral amygdalar nucleus	
Retrosplenial area dorsal part	
Interpeduncular nucleus	
Red nucleus	
Central lobule	
Retrosplenial area lateral agranular part	
Midbrain reticular nucleus retrorubral area	
Ventral tegmental area	
Midbrain reticular nucleus	
Superior colliculus motor related	
Basolateral amygdalar nucleus	
Pons motor related	
Anterior cingulate area ventral part	
Pedunculopontine nucleus	
Subparafascicular nucleus	
Claustrum	
Pons	
Superior colliculus sensory related	
Posterior hypothalamic nucleus	
Pontine reticular nucleus	
Medial geniculate complex	

Figure 1-1: Organization of the regions in the clustered correlation matrices for the saline and nicotine groups (Fig. 1B and C)

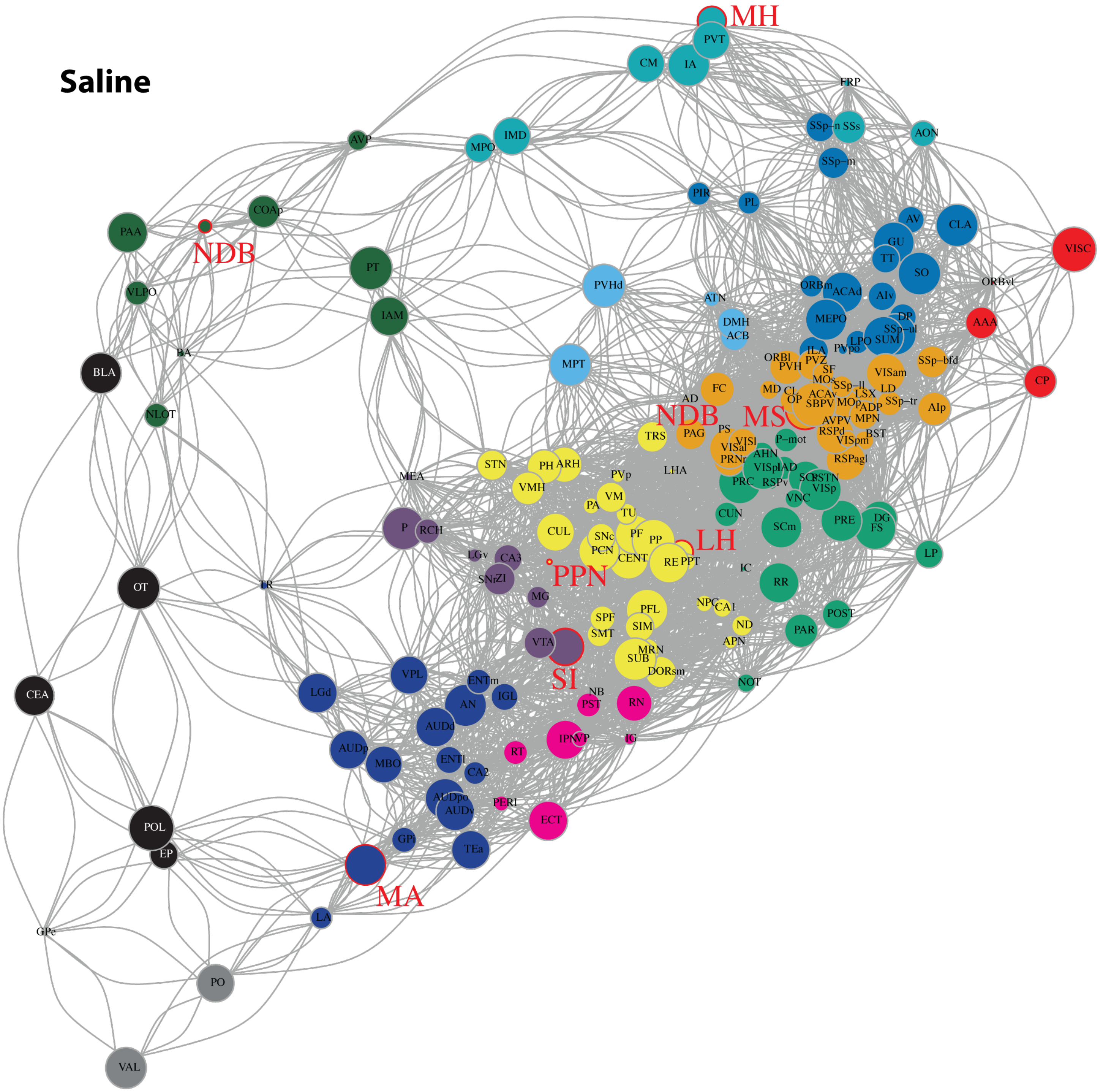

Figure 1-2: zoom and node label of Fig 1D

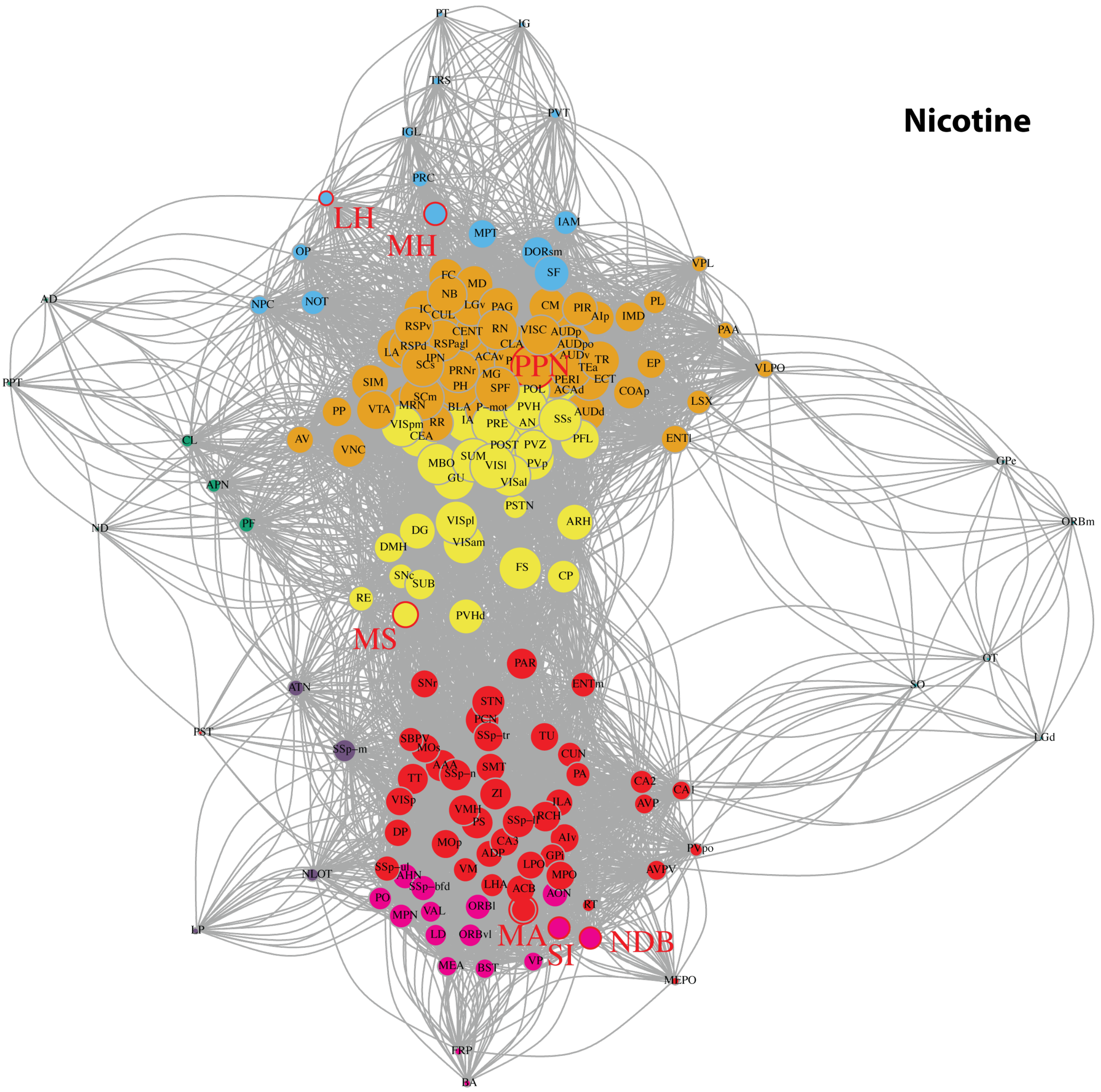

Figure 1-3: zoom and node labels of Fig 1E

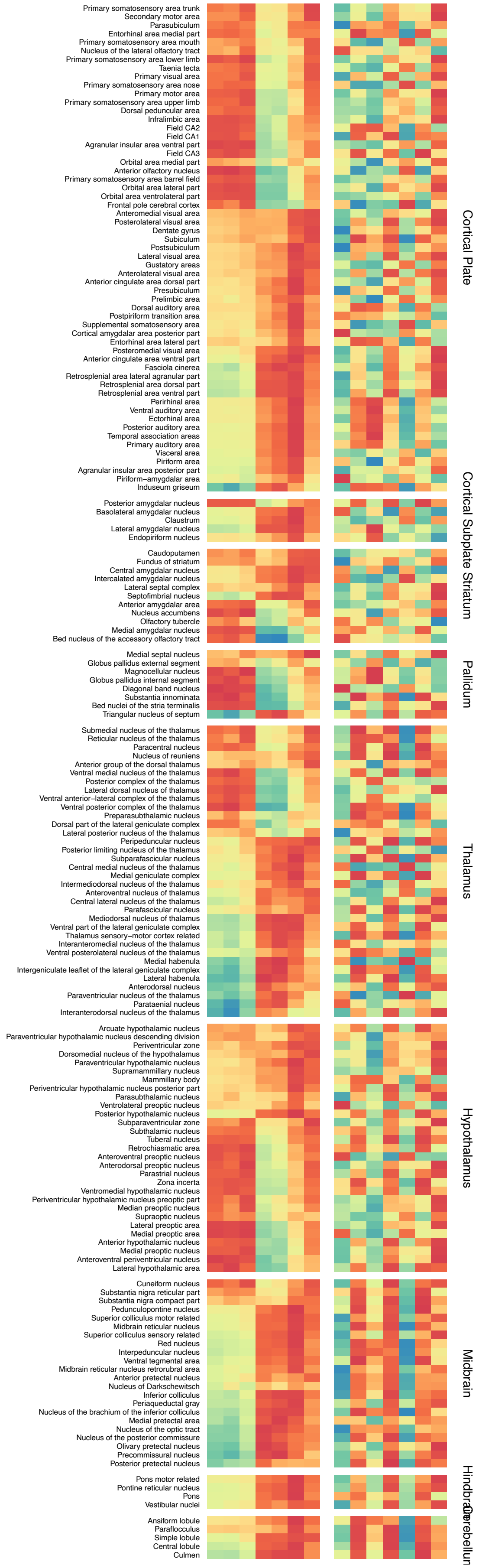

Figure 2-1: Organization of the regions in the correlation matrix separated by anatomical groups (Fig. 2E and 3D)

Table 1-1: Published Fos counts during nicotine withdrawal and saline controls

Region	Abb	Sal1	Sal2	Sal3	Sal4	Nic1	Nic2	Nic3	Nic4	Nic5
Agranular insular area posterior part	AIp	496	1083	1364	991	1195	278	549	1191	991
Agranular insular area ventral part	AIv	155	502	408	221	284	494	306	738	550
Ansiform lobule	AN	47	16	17	75	20	0	17	381	95
Anterior amygdalar area	AAA	66	88	102	79	92	154	154	286	181
Anterior cingulate area dorsal part	ACAd	294	2954	1008	506	798	176	553	2649	2315
Anterior cingulate area ventral part	ACAv	210	1947	816	1159	1040	95	1177	3819	4540
Anterior group of the dorsal thalamus	ATN	12	23	11	14	3	13	66	69	31
Anterior hypothalamic nucleus	AHN	100	407	187	445	53	1231	500	3262	544
Anterior olfactory nucleus	AON	96	308	587	47	212	892	245	1427	546
Anterior pretectal nucleus	APN	54	83	131	361	44	39	582	371	143
Anterodorsal nucleus	AD	19	126	17	64	13	4	213	25	25
Anterodorsal preoptic nucleus	ADP	1	12	7	7	8	17	13	24	23
Anterolateral visual area	VISal	38	215	57	210	94	33	99	479	157
Anteromedial visual area	VISam	51	1388	795	445	290	206	776	4010	1444
Anteroventral nucleus of thalamus	AV	36	64	60	37	36	23	160	146	91
Anteroventral periventricular nucleus	AVPV	0	46	12	22	20	120	9	284	113
Anteroventral preoptic nucleus	AVP	51	22	16	3	18	51	14	153	148
Arcuate hypothalamic nucleus	ARH	44	137	9	131	44	17	27	668	391
Basolateral amygdalar nucleus	BLA	700	354	458	266	393	130	403	771	481
Bed nuclei of the stria terminalis	BST	125	374	325	344	154	3701	449	3124	837
Bed nucleus of the accessory olfactory tract	BA	40	13	6	10	7	96	9	42	9
Caudoputamen	CP	5517	8131	12885	7919	10055	9691	10145	15966	15386
Central amygdalar nucleus	CEA	565	301	485	291	369	261	475	650	582
Central lateral nucleus of the thalamus	CL	7	19	11	14	8	5	217	78	44
Central lobule	CENT	74	129	52	340	115	0	111	494	230
Central medial nucleus of the thalamus	CM	21	29	19	9	16	0	5	22	86
Claustrum	CLA	255	568	654	271	454	142	402	683	618
Cortical amygdalar area posterior part	COAp	245	159	54	49	244	45	83	501	195
Culmen	CUL	78	180	16	518	95	0	116	176	290
Cuneiform nucleus	CUN	2	13	5	27	3	9	6	31	32
Dentate gyrus	DG	1305	2285	3007	3272	1420	1275	3347	6816	3709
Diagonal band nucleus	NDB	273	100	41	29	138	658	117	734	475
Dorsal auditory area	AUDd	2095	1282	1423	2322	2239	534	1004	5255	3344
Dorsal part of the lateral geniculate complex	LGd	116	9	15	62	89	78	12	146	76
Dorsal peduncular area	DP	16	169	123	42	19	115	89	388	79
Dorsal premammillary nucleus	PMd	34	10	1	7	0	0	2	149	9
Dorsomedial nucleus of the hypothalamus	DMH	116	454	118	184	33	31	285	859	213
Ectorhinal area	ECT	907	532	1188	1734	2011	262	796	3920	2407
Endopiriform nucleus	EP	811	657	793	735	1240	411	541	1623	1150
Entorhinal area lateral part	ENTl	1009	329	449	1367	1110	172	206	2762	1061
Entorhinal area medial part	ENTm	37	11	9	88	29	34	19	308	84

Fasciola cinerea	FC	5	83	6	28	15	0	24	23	93
Field CA1	CA1	1505	1937	1992	3435	2148	3146	1295	8391	3982
Field CA2	CA2	323	218	257	363	310	421	254	780	508
Field CA3	CA3	750	733	587	842	792	1703	1111	3420	1557
Frontal pole cerebral cortex	FRP	6	13	13	2	4	1081	86	151	457
Fundus of striatum	FS	16	45	73	84	40	33	51	211	115
Globus pallidus external segment	GPe	139	56	282	72	259	104	42	353	144
Globus pallidus internal segment	GPi	28	6	25	36	29	101	31	330	66
Gustatory areas	GU	426	1312	1030	471	651	501	858	1345	978
Induseum griseum	IG	6	3	11	43	47	0	11	1	55
Inferior colliculus	IC	119	502	398	2183	234	3	482	663	1742
Infralimbic area	ILA	121	1211	362	385	571	812	545	1451	965
Interanterodorsal nucleus of the thalamus	IAD	6	21	12	25	7	3	38	0	15
Interanteromedial nucleus of the thalamus	IAM	18	20	2	7	9	0	3	4	24
Intercalated amygdalar nucleus	IA	66	89	59	41	51	25	73	130	109
Intergeniculate leaflet of the lateral geniculate complex	IGL	41	23	22	64	22	0	33	4	88
Intermediodorsal nucleus of the thalamus	IMD	9	12	6	5	9	0	1	12	33
Interpeduncular nucleus	IPN	67	35	76	265	42	0	71	233	50
Lateral amygdalar nucleus	LA	594	328	702	649	747	213	946	1156	944
Lateral dorsal nucleus of thalamus	LD	59	97	91	85	48	537	160	830	177
Lateral habenula	LH	46	105	51	256	42	0	247	19	79
Lateral hypothalamic area	LHA	608	1240	556	1371	977	2807	923	6692	1552
Lateral posterior nucleus of the thalamus	LP	59	101	202	171	47	359	630	422	187
Lateral preoptic area	LPO	62	312	172	140	117	771	221	1717	745
Lateral septal complex	LSX	260	798	623	628	1303	584	607	1796	2138
Lateral visual area	VISl	64	233	89	222	33	10	50	307	110
Magnocellular nucleus	MA	22	16	22	23	1	78	6	112	109
Mammillary body	MBO	35	9	12	36	9	0	43	860	53
Medial amygdalar nucleus	MEA	591	409	144	518	167	947	328	873	388
Medial geniculate complex	MG	155	126	68	337	85	1	80	584	174
Medial habenula	MH	277	410	339	75	117	0	143	31	536
Medial preoptic area	MPO	296	359	202	180	156	1028	276	1969	981
Medial preoptic nucleus	MPN	1	157	49	49	11	349	97	470	181
Medial pretectal area	MPT	48	82	29	54	17	0	13	9	69
Medial septal nucleus	MS	7	69	25	42	36	50	108	166	166
Median preoptic nucleus	MEPO	4	136	26	19	16	66	24	62	193
Mediodorsal nucleus of thalamus	MD	60	189	80	127	161	6	149	173	313
Midbrain reticular nucleus	MRN	324	350	345	864	227	33	510	1200	561
Midbrain reticular nucleus retrorubral area	RR	17	50	77	172	43	4	46	168	34
Nucleus accumbens	ACB	288	954	302	454	214	1222	445	2066	1784
Nucleus of Darkschewitsch	ND	2	3	4	8	1	0	2	4	0
Nucleus of reunions	RE	42	65	42	130	29	32	132	401	68
Nucleus of the brachium of the inferior colliculus	NB	79	64	80	206	78	1	89	105	192
Nucleus of the lateral olfactory tract	NLOT	260	30	13	30	3	60	108	107	134

Nucleus of the optic tract	NOT	21	32	84	161	21	0	167	35	87
Nucleus of the posterior commissure	NPC	28	55	50	253	10	0	213	27	41
Olfactory tubercle	OT	1125	49	126	115	1021	565	142	2816	474
Olivary pretectal nucleus	OP	10	169	38	68	15	0	142	15	68
Orbital area lateral part	ORBl	22	791	59	139	44	852	167	1553	306
Orbital area medial part	ORBm	65	719	147	102	314	188	66	313	351
Orbital area ventrolateral part	ORBvl	25	371	1480	109	67	1068	252	1371	415
Parabigeminal nucleus	PBG	12	10	0	17	9	0	27	11	10
Paracentral nucleus	PCN	5	9	2	23	6	12	9	124	18
Parafascicular nucleus	PF	6	18	3	52	7	7	73	67	21
Paraflocculus	PFL	10	14	10	45	13	0	2	155	30
Parastrial nucleus	PS	3	20	4	15	1	21	11	111	29
Parasubiculum	PAR	10	18	41	69	8	10	8	440	38
Parasubthalamic nucleus	PSTN	18	41	34	47	17	5	10	70	11
Parataenial nucleus	PT	267	267	139	186	182	11	113	16	221
Paraventricular hypothalamic nucleus	PVH	38	405	85	135	101	21	98	467	399
Paraventricular hypothalamic nucleus descending division	PVHd	22	64	12	22	18	19	32	104	38
Paraventricular nucleus of the thalamus	PVT	600	884	864	95	689	0	31	9	1019
Pedunculopontine nucleus	PPN	22	23	16	31	11	0	8	47	32
Periaqueductal gray	PAG	172	636	171	503	333	0	195	857	595
Peripeduncular nucleus	PP	7	12	6	22	1	0	15	15	38
Perirhinal area	PERI	1122	691	1114	1648	1295	259	769	2652	1793
Periventricular hypothalamic nucleus posterior part	PVp	12	40	5	49	11	0	6	189	22
Periventricular hypothalamic nucleus preoptic part	PVpo	2	112	26	18	8	38	13	80	203
Periventricular zone	PVZ	7	208	41	50	37	19	46	171	226
Piriform area	PIR	3522	4826	3418	3316	5806	1158	2281	5829	5116
Piriform-amygdalar area	PAA	341	74	32	24	852	12	28	657	173
Pons	P	369	312	217	358	439	2	219	2845	1220
Pons motor related	P-mot	39	226	92	221	62	0	77	1433	273
Pontine reticular nucleus	PRNr	51	119	61	124	65	0	76	510	195
Posterior amygdalar nucleus	PA	21	43	9	61	22	36	19	133	64
Posterior auditory area	AUDpo	88	7	36	150	193	2	47	591	378
Posterior complex of the thalamus	PO	20	5	93	30	3	245	66	773	40
Posterior hypothalamic nucleus	PH	303	441	164	450	77	0	123	1122	384
Posterior limiting nucleus of the thalamus	POL	12	7	11	9	7	3	8	14	24
Posterior pretectal nucleus	PPT	3	10	4	38	3	0	89	14	2
Posterolateral visual area	VISpl	6	37	14	45	1	0	9	117	20
posteromedial visual area	VISpm	9	1431	442	760	156	22	852	3071	1680
Postpiriform transition area	TR	223	28	18	69	89	8	22	206	168
Postsubiculum	POST	33	94	342	584	139	59	154	494	308
Precommissural nucleus	PRC	29	72	41	85	26	0	23	6	44
Prelimbic area	PL	279	1451	368	211	559	250	308	595	1010
Preparasubthalamic nucleus	PST	6	5	6	12	3	3	3	22	1
Presubiculum	PRE	61	282	371	588	152	23	160	1080	423

Primary auditory area	AUDp	1664	647	819	1486	2230	202	771	3565	2952
Primary motor area	MOp	716	14331	5991	6321	2814	11439	7902	23397	11076
Primary somatosensory area barrel field	SSp-bfd	1286	3242	3670	2285	1966	7040	4277	10541	4580
Primary somatosensory area lower limb	SSp-ll	295	5333	2162	2104	1110	2750	1775	4944	3859
Primary somatosensory area mouth	SSp-m	1073	4813	2147	711	850	1896	2933	4947	2214
Primary somatosensory area nose	SSp-n	1465	3286	2158	948	1341	2354	2290	5771	2622
Primary somatosensory area trunk	SSp-tr	56	2415	1502	958	477	1125	1283	3297	2578
Primary somatosensory area upper limb	SSp-ul	688	5664	3453	1863	927	4609	3311	7481	4302
Primary visual area	VISp	103	1793	918	3118	196	1672	1656	7180	2810
Red nucleus	RN	24	19	30	109	48	0	35	151	60
Reticular nucleus of the thalamus	RT	90	22	65	401	45	150	126	198	381
Retrochiasmatic area	RCH	190	81	10	176	52	250	89	1099	550
Retrosplenial area dorsal part	RSPd	107	3384	1282	1977	505	0	2341	5927	2977
Retrosplenial area lateral agranular part	RSPagl	47	1668	829	2169	547	0	954	5342	1827
Retrosplenial area ventral part	RSPv	228	2604	901	4150	1273	6	2906	5708	3226
Secondary motor area	MOs	645	7208	2528	2831	1740	4008	5508	10852	6794
Septofimbrial nucleus	SF	13	186	54	75	97	19	53	78	139
Simple lobule	SIM	20	40	22	728	15	1	156	218	239
Subiculum	SUB	252	270	279	1280	244	277	849	1986	811
Submedial nucleus of the thalamus	SMT	7	7	5	28	2	11	10	41	33
Subparafascicular nucleus	SPF	73	81	62	180	59	6	60	269	197
Subparaventricular zone	SBPV	5	248	56	90	7	40	76	222	156
Substantia innominata	SI	177	170	163	208	176	941	148	1496	397
Substantia nigra compact part	SNc	21	34	11	66	12	8	31	246	20
Substantia nigra reticular part	SNr	86	59	26	117	22	45	106	1022	97
Subthalamic nucleus	STN	30	36	14	36	9	18	20	152	42
Superior colliculus motor related	SCm	253	1169	890	2795	414	44	995	2742	1486
Superior colliculus sensory related	SCs	48	1520	602	2794	244	3	387	1633	706
Supplemental somatosensory area	SSs	3479	5752	5353	2603	3489	1380	2529	6370	3864
Suprachiasmatic nucleus	SCH	0	184	14	23	81	80	79	157	493
Supramammillary nucleus	SUM	8	32	22	16	6	0	12	218	28
Supraoptic nucleus	SO	2	33	30	4	40	44	4	97	248
Taenia tecta	TT	151	755	474	177	194	478	513	1159	601
Temporal association areas	TEa	1914	788	2007	2818	3272	336	1457	6394	4439
Thalamus sensory-motor cortex related	DORsm	4	5	6	45	15	0	5	9	40
Triangular nucleus of septum	TRS	14	207	5	143	48	0	28	2	93
Tuberal nucleus	TU	78	167	50	257	94	171	94	1184	372
Ventral anterior-lateral complex of the thalamus	VAL	25	21	42	30	19	95	44	185	39
Ventral auditory area	AUDv	1953	1330	1815	2191	2663	381	1079	4339	3884
Ventral medial nucleus of the thalamus	VM	79	127	55	152	86	320	92	1094	166
Ventral part of the lateral geniculate complex	LGv	219	194	132	239	174	3	137	210	289
Ventral posterior complex of the thalamus	VP	38	24	39	102	36	435	44	634	81
Ventral posterolateral nucleus of the thalamus	VPL	104	31	30	126	64	17	21	44	48
Ventral tegmental area	VTA	199	135	94	495	83	1	438	1789	168

Ventrolateral preoptic nucleus	VLPO	67	8	4	1	76	22	18	84	93
Ventromedial hypothalamic nucleus	VMH	40	71	13	75	30	194	93	1267	159
Vestibular nuclei	VNC	20	90	57	137	7	0	152	428	32
Visceral area	VISC	866	1114	1762	974	1073	248	655	1578	1124
Zona incerta	ZI	348	277	151	464	227	520	360	1296	559

Table 4-1: List of significantly correlated genes (Fig. 4C with false discovery rate. (FDR) at 5%)

	set	n	cor	p	FDR	Bonferroni
175	Arl6	156	0.49504278	5.05E-11	7.41E-07	1.29E-06
1001	Mpo	156	0.49366662	5.82E-11	7.41E-07	1.48E-06
1066	Nek7	160	-0.4641837	6.31E-10	4.22E-06	1.61E-05
990	Mmp15	158	0.46347895	8.65E-10	4.22E-06	2.20E-05
299	Cd8b1	154	0.46750657	9.79E-10	4.22E-06	2.49E-05
1519	Sycn	156	0.46365193	1.09E-09	4.22E-06	2.78E-05
773	Igf2bp1	156	0.46233287	1.23E-09	4.22E-06	3.14E-05
564	Fam53a	156	0.46153767	1.33E-09	4.22E-06	3.38E-05
1036	Mypn	155	0.45112842	3.84E-09	1.09E-05	9.78E-05
1664	Vars	155	0.4499021	4.28E-09	1.09E-05	0.00010898
382	Col2a1	159	0.44360592	4.73E-09	1.10E-05	0.00012049
1135	Orm1	152	0.44975281	6.14E-09	1.30E-05	0.00015645
1508	Styxl1	156	0.44113478	8.22E-09	1.52E-05	0.00020935
580	Fbxl4	151	0.44748325	8.38E-09	1.52E-05	0.0002135
1275	Rab13	155	0.44131491	9.05E-09	1.54E-05	0.0002304
876	Lhx8	159	0.43277691	1.22E-08	1.94E-05	0.00031011
541	Etfa	160	-0.4281883	1.62E-08	2.43E-05	0.00041296
1516	Suv420h2	155	0.43266103	1.88E-08	2.65E-05	0.00047993
153	Appbp2	156	0.43080749	1.98E-08	2.65E-05	0.00050392
369	Cmah	158	0.42577597	2.44E-08	3.09E-05	0.00062249
1025	Mucl1	156	0.42778783	2.54E-08	3.09E-05	0.00064791
1426	Slc2a1	158	-0.4215244	3.48E-08	4.02E-05	0.000885
283	Ccdc88b	156	0.42201815	4.09E-08	4.52E-05	0.00104025
1218	Pmaip1	158	0.41892355	4.30E-08	4.56E-05	0.00109494
1675	Vps37c	151	0.42695566	4.58E-08	4.66E-05	0.00116599
797	Isl1	160	0.41514068	4.81E-08	4.71E-05	0.00122377
1039	N4bp1	158	0.41698715	5.03E-08	4.75E-05	0.00128148
1072	Ngfr	158	0.41632383	5.31E-08	4.83E-05	0.00135213
871	Lgals8	156	0.4176704	5.80E-08	5.10E-05	0.0014776
1229	Ppih	154	0.41898946	6.37E-08	5.13E-05	0.00162203
1198	Pik3r3	160	-0.4116379	6.38E-08	5.13E-05	0.00162549
1413	Slc17a9	158	0.41392053	6.44E-08	5.13E-05	0.00164072
653	Gm13420	160	0.4053327	1.06E-07	8.14E-05	0.0026877
58	9530077C05Rik	160	0.40358046	1.21E-07	8.85E-05	0.00308517
1700	Xbp1	160	-0.4035334	1.22E-07	8.85E-05	0.00309659
1407	Slc15a2	154	0.40998692	1.29E-07	9.12E-05	0.0032832
475	Dph6	156	0.40541197	1.52E-07	0.00010463	0.00387137
863	Lect1	156	-0.4044905	1.63E-07	0.00010936	0.00415564
1626	Ttll12	152	0.40508863	2.26E-07	0.00014582	0.00575088
448	Depdc7	155	0.40119397	2.30E-07	0.00014582	0.00585341
1242	Pramel7	155	0.40091328	2.35E-07	0.00014582	0.00597869

1455	Smpd2	156	0.39917528	2.45E-07	0.00014828	0.00622794
1080	Nlrx1	156	0.39792642	2.69E-07	0.00015762	0.00684204
932	Luc7l	152	0.40215621	2.81E-07	0.00015762	0.00715779
873	Lhx6	156	0.39719322	2.84E-07	0.00015762	0.00722912
1240	Ppp4c	156	0.39715417	2.85E-07	0.00015762	0.00725031
1363	Sass6	155	0.39790404	2.94E-07	0.00015943	0.00749328
704	Gstk1	160	-0.3904065	3.33E-07	0.00017165	0.00848676
213	Baat	152	0.39983062	3.34E-07	0.00017165	0.00850178
1537	Tap1	155	0.39608111	3.37E-07	0.00017165	0.0085824
527	Epas1	158	-0.3918164	3.56E-07	0.00017784	0.00906969
469	Dnajb4	152	0.39827977	3.74E-07	0.00018324	0.00952862
1601	Tnfrsf11b	156	0.39288985	3.91E-07	0.00018657	0.00995878
953	Mapkap1	156	0.39261405	3.99E-07	0.00018657	0.01016377
726	Hbb-b2	160	-0.3878695	4.03E-07	0.00018657	0.01026138
101	Adm	153	0.39481773	4.41E-07	0.0002006	0.01123368
521	Enox2	154	0.39218022	4.90E-07	0.00021891	0.01247804
786	Insl5	156	0.38927721	5.10E-07	0.0002239	0.01298625
1405	Slc14a1	157	0.38775626	5.24E-07	0.00022597	0.0133325
1053	Ncdn	160	-0.3814801	6.46E-07	0.00027396	0.0164378
858	Lap3	156	0.38364031	7.67E-07	0.0003192	0.01952683
1216	Pltp	160	-0.3789255	7.77E-07	0.0003192	0.01979039
1589	Tmem183a	156	0.38279934	8.14E-07	0.00032918	0.02073855
1131	Oog3	155	0.38358576	8.36E-07	0.00033268	0.02129177
182	Art4	156	0.3820081	8.62E-07	0.0003376	0.02194382
969	Med27	125	0.42265788	9.10E-07	0.00035122	0.0231808
473	Dnase2a	155	0.38207512	9.31E-07	0.00035379	0.02370411
780	Il17rb	155	0.38145582	9.73E-07	0.00035493	0.02476652
1073	Nhp2l1	152	0.38496076	9.73E-07	0.00035493	0.02477913
1111	Oas1c	155	0.38141091	9.76E-07	0.00035493	0.0248453
791	Irs4	160	0.37506123	1.03E-06	0.00036289	0.02612755
14	2310022A10Rik	155	0.38033535	1.05E-06	0.00036289	0.02680506
897	LOC433005	159	-0.3756483	1.06E-06	0.00036289	0.02711357
117	Akr1b8	151	0.38481094	1.07E-06	0.00036289	0.02721494
1442	Slc6a20b	160	-0.3744887	1.07E-06	0.00036289	0.02721698
711	H2-D1	159	-0.375018	1.11E-06	0.0003731	0.02835558
271	Cbx5	154	0.37986645	1.18E-06	0.00038605	0.03004144
1237	Ppp1r3d	156	-0.3775222	1.18E-06	0.00038605	0.03014353
1334	Rnpc3	150	0.3842794	1.21E-06	0.00038605	0.03068622
1340	Rps13	154	0.37947035	1.21E-06	0.00038605	0.03088437
1403	Slamf7	152	0.38136536	1.25E-06	0.00039315	0.03184478
144	Ap4m1	156	0.37589037	1.33E-06	0.00041137	0.03379438
511	Eif6	156	0.37574311	1.34E-06	0.00041137	0.03414381
364	Clk1	156	-0.3753641	1.38E-06	0.00041737	0.0350589
1553	Tcl1b1	156	0.37428457	1.48E-06	0.00043999	0.03779545
1307	Rgs5	158	-0.3720293	1.49E-06	0.00043999	0.0378395

1287	Rbbp6	155	0.37494314	1.53E-06	0.00044547	0.03906215
746	Hook3	156	0.37375864	1.54E-06	0.00044547	0.03920115
108	Ahsg	155	0.37465626	1.56E-06	0.0004477	0.03984509
841	Klhl15	156	0.37333569	1.59E-06	0.0004477	0.04036755
427	Cyp26a1	156	0.37320293	1.60E-06	0.0004477	0.04074044
45	5031414D18Rik	158	0.37072944	1.63E-06	0.0004503	0.04142729
1691	Wisp1	159	0.36743558	1.89E-06	0.00051873	0.0482423
461	Dlx3	160	-0.3658268	1.96E-06	0.00053216	0.05002259
120	Aldh1l1	160	-0.365581	2.00E-06	0.00053559	0.05088107
614	Gab1	159	-0.3653867	2.18E-06	0.00057628	0.05556359
1402	Six3os1	160	0.36421849	2.20E-06	0.00057628	0.05589916
949	Map1lc3b	160	-0.363035	2.38E-06	0.00061632	0.0606366
1138	Ostc	158	0.36510853	2.40E-06	0.00061632	0.06101611
380	Cntrl	159	-0.3633043	2.52E-06	0.00064081	0.06408054
1090	Nrg4	158	0.36412586	2.56E-06	0.00064595	0.06524058
994	Mmp9	156	0.36605425	2.61E-06	0.00065141	0.06644378
484	Duoxa1	156	0.36588997	2.64E-06	0.00065229	0.06718564
1714	Zbbx	158	-0.3632251	2.72E-06	0.00065428	0.06935612
1078	Nit2	158	0.36321758	2.73E-06	0.00065428	0.06939133
1141	Otx2	154	-0.3673893	2.77E-06	0.00065428	0.07059471
501	Efhd1	155	0.36623403	2.78E-06	0.00065428	0.0707554
358	Cldn5	160	-0.3607449	2.78E-06	0.00065428	0.07090911
1178	Peg10	150	0.37167401	2.83E-06	0.00065428	0.07204334
1179	Peg10	160	0.36050174	2.83E-06	0.00065428	0.07209217
988	Mmp11	151	0.37039008	2.85E-06	0.00065428	0.07262464
1397	Shroom1	158	0.3621379	2.93E-06	0.00066653	0.07465192
958	Matn4	155	0.36440426	3.14E-06	0.00070784	0.07998631
396	Cpa1	156	0.3627855	3.25E-06	0.00072321	0.08277586
388	Colec11	160	0.35810725	3.33E-06	0.00072321	0.08478805
794	Irx2	160	-0.3580114	3.35E-06	0.00072321	0.08533807
1647	Uck2	158	0.36015014	3.35E-06	0.00072321	0.08534285
1021	Mttp	156	0.36220816	3.38E-06	0.00072321	0.08603074
761	Htr3b	156	0.36220269	3.38E-06	0.00072321	0.08606216
634	Gatsl3	160	0.35763661	3.44E-06	0.00072934	0.08752138
225	Bcs1l	155	0.3626769	3.52E-06	0.00073944	0.08974111
30	3830406C13Rik	160	-0.3571868	3.54E-06	0.00073944	0.09021192
1378	Serpina1d	158	0.35900162	3.62E-06	0.00074639	0.09216682
659	Gm4912	159	-0.3578494	3.64E-06	0.00074639	0.09266571
865	Lefty1	155	0.36209099	3.66E-06	0.00074639	0.093299
494	Ebf4	160	0.3564076	3.73E-06	0.00075338	0.09505909
751	Hsd17b13	159	0.35737131	3.76E-06	0.00075338	0.0956796
325	Celf6	155	0.36153085	3.80E-06	0.00075645	0.09682525
408	Crisp1	155	0.36056209	4.05E-06	0.00080019	0.1032244
414	Ctage5	156	0.35773751	4.54E-06	0.00088187	0.11568011
735	Hiatl1	158	-0.3555484	4.55E-06	0.00088187	0.1159421

1532	Tacr2	153	-0.3609332	4.58E-06	0.00088187	0.11652135
146	Apbb1	160	-0.3532583	4.61E-06	0.00088187	0.11728832
723	Hbb	160	-0.3530075	4.68E-06	0.00088956	0.11925596
1003	Mpzl2	155	0.35825853	4.72E-06	0.00088956	0.12009022
609	Fzd7	157	-0.3559162	4.77E-06	0.00089125	0.12144656
1583	Tmem125	158	0.35476403	4.80E-06	0.00089125	0.12210087
1341	Rps15	155	0.3576056	4.92E-06	0.00090817	0.12532683
478	Drd3	155	0.35727622	5.03E-06	0.00091939	0.12804997
852	Kti12	158	0.35396245	5.05E-06	0.00091939	0.12871425
618	Gabrq	160	0.35154967	5.16E-06	0.00092568	0.13132846
160	Aqp4	160	-0.3515361	5.16E-06	0.00092568	0.13144605
1384	Setx	159	-0.3523856	5.23E-06	0.00093132	0.13317901
1679	Vtn	160	-0.3510838	5.32E-06	0.00094045	0.13542447
1172	Pdha1	160	-0.3509534	5.36E-06	0.00094202	0.13659284
532	Epha10	160	-0.3507756	5.43E-06	0.00094593	0.13820122
316	Cdk20	152	0.35932499	5.46E-06	0.00094593	0.13905154
810	Jmjd8	155	0.35584601	5.52E-06	0.00094958	0.14053847
276	Ccdc155	152	0.35899978	5.58E-06	0.00095298	0.14199457
700	Grwd1	152	0.35873069	5.67E-06	0.00096316	0.14447428
159	Aqp4	160	-0.3499692	5.72E-06	0.00096505	0.14572278
70	Abcc4	159	-0.3500914	6.08E-06	0.00101277	0.15477739
269	Cbwd1	158	0.35105851	6.11E-06	0.00101277	0.15563099
627	Galnt10	149	0.36093301	6.12E-06	0.00101277	0.15596668
1631	Tubb2b	146	-0.3638483	6.35E-06	0.00103883	0.16174735
1484	Spc25	155	0.35364286	6.36E-06	0.00103883	0.16205816
1689	Wee2	152	0.35603084	6.74E-06	0.00109383	0.17173165
1367	Scara5	155	0.35250753	6.85E-06	0.00110337	0.17433182
1254	Prss58	158	-0.3490415	6.97E-06	0.00111558	0.17737771
118	Akr1c13	153	0.35421376	7.06E-06	0.00112311	0.17969724
970	Med7	155	0.35160905	7.25E-06	0.00114698	0.18466369
1505	Stoml2	160	-0.3459877	7.42E-06	0.00116607	0.18890274
116	Aknaos	156	0.34981853	7.59E-06	0.00118617	0.19334652
1467	Snx16	144	-0.3629588	7.79E-06	0.00120853	0.19826617
1599	Tmem9	158	0.34722429	7.83E-06	0.00120853	0.19940777
1678	Vtn	160	-0.3449702	7.92E-06	0.00121532	0.20174268
1116	Odf3b	159	-0.345688	8.08E-06	0.00122769	0.20585793
552	Fam122c	156	0.34871698	8.15E-06	0.00122769	0.2074406
1269	Pwp1	155	0.34978269	8.15E-06	0.00122769	0.2074792
548	F2	158	0.346335	8.29E-06	0.00124182	0.21110973
138	Anxa13	156	0.34819259	8.42E-06	0.00125431	0.21448662
1358	S100b	158	-0.3455673	8.71E-06	0.00127887	0.22173184
829	Kctd9	152	0.35195746	8.73E-06	0.00127887	0.22223196
1721	Zcchc5	158	0.34551142	8.74E-06	0.00127887	0.22252421
190	Atf3	155	0.3483847	8.90E-06	0.00129554	0.2267194
747	Hoxa10	160	0.34299594	8.99E-06	0.00130141	0.22904771

790	Iqub	160	-0.3428216	9.10E-06	0.00130859	0.23162004
918	LOC545854	160	-0.342656	9.19E-06	0.00131511	0.23408897
720	Hba-a1	155	-0.3477245	9.28E-06	0.00132057	0.23638126
1257	Psg16	159	-0.3432965	9.42E-06	0.00133183	0.23991398
694	Grin3b	159	0.34321895	9.47E-06	0.00133183	0.24110292
1336	Rpgrip1	160	-0.3420913	9.53E-06	0.00133183	0.24269639
1228	Ppargc1b	156	-0.3461776	9.57E-06	0.00133183	0.24372501
1289	Rbm17	156	0.34609022	9.62E-06	0.00133193	0.245075
976	Mettl22	155	0.34673305	9.88E-06	0.00135654	0.25162587
728	Hck	156	0.345629	9.91E-06	0.00135654	0.25231632
538	Esam	155	-0.3457181	1.05E-05	0.00143418	0.26819135
849	Kremen1	154	-0.3463063	1.09E-05	0.00146326	0.27635013
833	Kif15	156	0.34417133	1.09E-05	0.00146326	0.27655583
1404	Slc10a4	159	0.34053459	1.12E-05	0.00150457	0.2858686
848	Krcc1	156	0.3434767	1.13E-05	0.00151239	0.28886614
219	Bcan	160	-0.3388731	1.17E-05	0.00154594	0.2977498
1214	Plscr1	156	0.3429595	1.17E-05	0.00154594	0.2983666
1434	Slc3a1	158	0.34075903	1.18E-05	0.00154599	0.30066719
286	Ccl22	155	0.34384702	1.18E-05	0.00154599	0.30146807
1321	Riok2	154	0.34473305	1.20E-05	0.00155508	0.30479586
133	Amph	160	-0.33819	1.22E-05	0.001578	0.31086504
287	Ccnb1	155	0.34268844	1.27E-05	0.00163631	0.32398979
824	Kcnmb4os2	159	-0.3382798	1.29E-05	0.00165028	0.32943433
502	Ehmt2	159	-0.3382497	1.30E-05	0.00165028	0.33005552
603	Frg1	158	0.33860138	1.35E-05	0.00171211	0.34413415
303	Cdc20	156	0.34045393	1.37E-05	0.00172635	0.34872251
721	Hba-a1	160	-0.3360844	1.39E-05	0.00174783	0.35480931
464	Dmrtc1c2	160	0.3352056	1.47E-05	0.00183202	0.37483475
1499	Stambpl1	155	0.34029858	1.47E-05	0.00183202	0.37556337
1088	Nr2c1	159	0.33570116	1.52E-05	0.0018783	0.38692887
65	Aaas	156	0.338597	1.54E-05	0.00188943	0.39111127
1038	Mzt2	155	0.33938583	1.56E-05	0.00190978	0.39723327
1423	Slc25a5	160	-0.3339135	1.60E-05	0.00194366	0.40622472
769	Ifi202b	153	0.34091181	1.62E-05	0.00196033	0.41166939
703	Gsta3	155	0.33854997	1.64E-05	0.00197913	0.41810833
1634	Txndc5	156	0.33745393	1.65E-05	0.00197913	0.41957564
1746	Zfx	158	-0.3352756	1.66E-05	0.00198339	0.42292892
1069	Ngb	160	0.33320603	1.67E-05	0.00198339	0.42444591
449	Dera	155	0.33783001	1.72E-05	0.00201347	0.43691436
103	Agap3	160	-0.3327282	1.72E-05	0.00201347	0.43718748
1108	Nxph1	160	-0.3326799	1.72E-05	0.00201347	0.43849538
1464	Snhg7	160	-0.332652	1.72E-05	0.00201347	0.43925179
266	Cartpt	158	0.33454854	1.74E-05	0.00201347	0.44228662
61	A830019P07Rik	156	-0.3365683	1.74E-05	0.00201347	0.44296243
1005	Mrfap1	155	-0.3372501	1.78E-05	0.00204763	0.45264045

1130	Onecut1	158	-0.3341025	1.79E-05	0.00204763	0.45457348
1019	Mtif2	151	0.34129513	1.80E-05	0.00205369	0.45797231
367	Cltc	159	-0.3328698	1.81E-05	0.00205541	0.4609203
161	Ar	160	0.33181775	1.82E-05	0.00205541	0.46246808
218	BC051665	155	0.33671668	1.84E-05	0.00206072	0.46757425
466	Dnah12	154	-0.3376281	1.85E-05	0.00206072	0.47133669
1318	Rin2	156	0.33546693	1.86E-05	0.00206072	0.47376119
666	Gm953	160	-0.3313738	1.87E-05	0.00206072	0.47529102
914	LOC545133	159	-0.3323517	1.87E-05	0.00206072	0.47582994
946	Magt1	155	0.33637855	1.87E-05	0.00206072	0.47728078
363	Clic4	160	-0.3312785	1.88E-05	0.00206072	0.47808678
1388	Sfxn2	155	0.33600079	1.92E-05	0.00209592	0.4883492
265	Cartpt	160	0.3303326	1.99E-05	0.00216117	0.50668998
435	Dbr1	160	-0.3302945	1.99E-05	0.00216117	0.50787494
1032	Myg1	155	0.33510936	2.02E-05	0.00218405	0.51543569
779	Il17b	152	0.33806444	2.05E-05	0.00219276	0.52140727
130	Alppl2	157	0.33281534	2.05E-05	0.00219276	0.52313285
1474	Son	160	-0.3297822	2.06E-05	0.00219276	0.52407032
250	C6	158	0.33155897	2.09E-05	0.0022126	0.53102448
892	LOC432692	159	-0.3302197	2.13E-05	0.00224938	0.54210142
354	Cisd2	152	0.33671641	2.22E-05	0.00233544	0.56517618
1597	Tmem38b	157	0.33097364	2.30E-05	0.0024038	0.5848386
66	Aadac	156	0.33194046	2.30E-05	0.0024038	0.58652789
1010	Mrps23	156	0.33144013	2.37E-05	0.00246455	0.6044403
1187	Pglyrp2	155	0.33241208	2.38E-05	0.00246455	0.60627835
24	2610305D13Rik	159	-0.3281774	2.41E-05	0.0024845	0.61367146
1432	Slc38a2	156	-0.3308047	2.47E-05	0.00253199	0.62793286
1711	Ywhae	160	-0.3264423	2.52E-05	0.00257906	0.64218614
1742	Zfp819	152	0.33447791	2.54E-05	0.00258236	0.64558891
1577	Tmbim6	160	-0.3259787	2.59E-05	0.00263124	0.66044012
148	Aplf	158	0.32775568	2.62E-05	0.00265187	0.66827144
945	Magel2	160	0.32536421	2.69E-05	0.00270904	0.68538755
72	Abhd4	152	0.33324482	2.73E-05	0.00273373	0.69436853
319	Cdr2	160	-0.3247281	2.80E-05	0.00278463	0.71214628
948	Map1b	158	-0.326665	2.80E-05	0.00278463	0.71341101
1201	Piwil4	156	-0.3286149	2.81E-05	0.00278463	0.71564902
75	Acacb	160	-0.3245211	2.83E-05	0.00279483	0.72106531
1696	Wrn	158	-0.3259243	2.93E-05	0.00287866	0.74568922
1610	Tpi1	157	-0.3268568	2.94E-05	0.00287866	0.74845267
315	Cdhr4	158	-0.3251614	3.06E-05	0.0029899	0.78036405
1512	Sult1c1	156	0.32694305	3.10E-05	0.00300957	0.7902504
203	Atp6v0a4	156	0.32685809	3.12E-05	0.00300957	0.79423021
1390	Sgk1	158	-0.324859	3.12E-05	0.00300957	0.79452537
1278	Rad23b	155	-0.3275842	3.17E-05	0.0030477	0.80763991
916	LOC545666	159	-0.323468	3.20E-05	0.00306042	0.81407232

238	Bpifa1	156	0.32625132	3.23E-05	0.00308151	0.82320716
1186	Pgk1	159	-0.323193	3.25E-05	0.00308151	0.82750128
1124	Olfr536	158	-0.324075	3.27E-05	0.00308151	0.83237397
1046	Naprt	156	0.32602067	3.28E-05	0.00308151	0.83448054
1004	Mrc1	160	-0.3220264	3.29E-05	0.00308151	0.83714115
714	H2-T23	154	0.32790279	3.30E-05	0.00308151	0.84151653
127	Alkbh6	160	-0.3219179	3.31E-05	0.00308151	0.84256874
1144	P2ry10	155	0.3268289	3.32E-05	0.00308151	0.84433452
1176	Pdyn	156	0.32573211	3.33E-05	0.00308651	0.8487895
1734	Zfp608	155	-0.3265879	3.36E-05	0.00310279	0.85637076
1374	Sema3f	160	-0.3212331	3.45E-05	0.00316821	0.8775936
803	Itm2a	160	-0.3210787	3.48E-05	0.00318589	0.88567853
1127	Olfr70	156	0.32482423	3.52E-05	0.00320906	0.89532756
498	Efcab14	159	-0.3217619	3.54E-05	0.00321713	0.90079704
366	Cln3	156	0.32438833	3.61E-05	0.00326874	0.91851518
1479	Sox7	158	-0.3219126	3.71E-05	0.00335131	0.94574332
1304	Rfx5	158	0.3218645	3.72E-05	0.00335131	0.94842115
1205	Pla2g7	160	-0.3198429	3.74E-05	0.00335548	0.9529574
246	C1ql4	160	0.31976346	3.76E-05	0.00335944	0.95744001
1097	Nts	159	0.32060016	3.79E-05	0.00337323	0.96474307
878	Lingo2	160	-0.3194246	3.84E-05	0.00340346	0.97679352
713	H2-T22	155	0.32411172	3.89E-05	0.00343652	0.98971708
121	Aldh1l2	154	0.3250546	3.90E-05	0.00343787	0.99354453
1535	Taok2	157	-0.3218108	3.96E-05	0.00347523	1
657	Gm454	158	-0.3204303	4.05E-05	0.00354528	1
917	LOC545780	158	0.32032161	4.08E-05	0.00355049	1
202	Atp6v0a1	160	-0.3183556	4.09E-05	0.00355049	1
641	Ghitm	160	-0.3182454	4.11E-05	0.00355968	1
712	H2-M3	155	0.3230921	4.12E-05	0.00355968	1
894	LOC432762	159	0.31863852	4.25E-05	0.0036571	1
513	Elovl1	160	-0.3173613	4.33E-05	0.00368481	1
1392	Sgpp1	156	0.32112732	4.36E-05	0.00368481	1
1148	Pacrgl	156	0.32105951	4.38E-05	0.00368481	1
709	Gys1	154	0.32304983	4.38E-05	0.00368481	1
152	App	160	-0.3171586	4.38E-05	0.00368481	1
1409	Slc16a4	160	-0.3171264	4.39E-05	0.00368481	1
491	Eaf2	156	0.32086676	4.43E-05	0.00368481	1
1140	Otx2	160	-0.3169665	4.43E-05	0.00368481	1
1252	Prpf31	115	0.37101987	4.47E-05	0.00368481	1
9	1810022K09Rik	159	-0.3177713	4.47E-05	0.00368481	1
925	Lrp8	160	-0.3167753	4.48E-05	0.00368481	1
95	Adamts3	160	-0.3167581	4.49E-05	0.00368481	1
339	Chad	156	0.32058876	4.50E-05	0.00368481	1
616	Gabrg1	159	0.31760955	4.51E-05	0.00368481	1
997	Mon1b	158	-0.3185663	4.52E-05	0.00368481	1

1412	Slc17a2	155	0.32149782	4.52E-05	0.00368481	1
1076	Nipsnap3b	152	0.32444798	4.54E-05	0.00368481	1
1050	Nbl1	160	-0.3164923	4.56E-05	0.00368481	1
1625	Ttc28	159	-0.3174436	4.56E-05	0.00368481	1
1680	Vwa1	160	-0.316209	4.63E-05	0.00372747	1
1028	Musk	156	-0.3200583	4.64E-05	0.00372747	1
227	Bend6	155	-0.320935	4.67E-05	0.00374042	1
493	Ebf1	158	-0.3177683	4.73E-05	0.00376674	1
1454	Smim3	158	0.31775897	4.73E-05	0.00376674	1
1132	Oprm1	157	-0.3186006	4.77E-05	0.00376782	1
1325	Rlbp1	152	0.32358022	4.77E-05	0.00376782	1
622	Gal	155	0.3205368	4.78E-05	0.00376782	1
596	Fndc8	159	-0.3165375	4.80E-05	0.00377634	1
528	Epb4.1l4a	158	-0.3174332	4.82E-05	0.00377945	1
474	Dopey1	158	-0.3173005	4.86E-05	0.0037892	1
403	Crabp2	155	0.32022395	4.87E-05	0.0037892	1
1098	Nts	160	0.3148103	5.03E-05	0.00390172	1
1417	Slc22a20	158	0.3164072	5.12E-05	0.0039499	1
499	Efemp2	160	-0.3143831	5.15E-05	0.0039499	1
886	LOC432444	159	-0.315281	5.17E-05	0.0039499	1
59	A330041J22Rik	158	-0.3162339	5.17E-05	0.0039499	1
1408	Slc16a10	154	0.32014992	5.17E-05	0.0039499	1
1248	Prl2c2	156	0.3181428	5.18E-05	0.0039499	1
1430	Slc35b2	159	0.31513268	5.21E-05	0.00396199	1
1524	Sypl	160	-0.3140501	5.25E-05	0.00398049	1
1410	Slc16a4	156	0.31715447	5.48E-05	0.00414257	1
1168	Pde7b	159	0.31402863	5.55E-05	0.00418498	1
206	Atp6v1g2	158	-0.3149229	5.57E-05	0.00418754	1
1209	Plekhh3	156	0.31670682	5.62E-05	0.00421231	1
263	Car9	158	-0.3144676	5.72E-05	0.0042731	1
680	Gpr149	158	0.31436873	5.75E-05	0.0042848	1
490	E2f6	160	-0.3123796	5.78E-05	0.0042941	1
729	Hdgf	160	-0.3121477	5.86E-05	0.00433913	1
1736	Zfp639	145	-0.3271101	5.92E-05	0.00437117	1
1372	Sds	149	0.32266682	5.99E-05	0.00440537	1
681	Gpr151	156	-0.3153304	6.08E-05	0.00446362	1
885	LOC381597	159	-0.3123016	6.13E-05	0.00448816	1
1740	Zfp763	152	0.31870645	6.29E-05	0.00458852	1
234	Bmp5	160	-0.3107524	6.35E-05	0.0046202	1
1606	Tor1b	156	0.31433888	6.43E-05	0.00466781	1
468	Dnah6	159	-0.3113105	6.49E-05	0.00467041	1
718	Haus5	158	-0.3122566	6.49E-05	0.00467041	1
1580	Tmem106c	155	0.31500574	6.55E-05	0.00467041	1
27	2810043G22Rik	156	-0.3139423	6.58E-05	0.00467041	1
839	Klhdc7a	158	-0.3120122	6.58E-05	0.00467041	1

855	Lair1	155	0.31488465	6.59E-05	0.00467041	1
1041	Nadsyn1	155	0.31487944	6.59E-05	0.00467041	1
87	Acp2	156	0.31386504	6.61E-05	0.00467041	1
1365	Scaf11	156	-0.3137997	6.63E-05	0.00467041	1
1648	Ufsp1	159	0.31087431	6.65E-05	0.00467041	1
1024	Mucl1	155	0.31465314	6.68E-05	0.00467041	1
132	Amd1	158	-0.311721	6.69E-05	0.00467041	1
1190	Pgr15l	160	0.30981926	6.70E-05	0.00467041	1
425	Cyhr1	160	-0.3098139	6.70E-05	0.00467041	1
1439	Slc5a11	151	0.31855068	6.71E-05	0.00467041	1
1380	Serpinb12	159	-0.3104776	6.81E-05	0.00471811	1
722	Hbb	147	-0.3224106	6.82E-05	0.00471811	1
1719	Zc3h12c	156	-0.3132569	6.84E-05	0.00471982	1
584	Fbxo8	155	0.31402379	6.92E-05	0.00475184	1
1639	Uba2	159	-0.3101768	6.92E-05	0.00475184	1
1339	Rpp25	160	-0.3087661	7.11E-05	0.00486838	1
311	Cdh5	158	-0.3105732	7.14E-05	0.00487558	1
1556	Tdgf1	155	0.31324509	7.23E-05	0.00492123	1
503	Ei24	160	-0.3083296	7.29E-05	0.00492601	1
268	Cbln2	160	-0.3082921	7.31E-05	0.00492601	1
1246	Prkab1	152	0.31597549	7.32E-05	0.00492601	1
1271	Qpct	156	0.31202599	7.33E-05	0.00492601	1
458	Diras1	160	-0.3082031	7.34E-05	0.00492601	1
1260	Psmc6	158	-0.3100616	7.35E-05	0.00492601	1
1249	Prl4a1	156	0.31164699	7.49E-05	0.00498492	1
1354	Rwdd2b	155	0.3125863	7.50E-05	0.00498492	1
774	Igf2bp3	155	0.31255648	7.51E-05	0.00498492	1
1438	Slc51a	155	0.31254341	7.52E-05	0.00498492	1
1502	Stat5b	156	0.31149303	7.55E-05	0.00499486	1
762	Htr6	147	0.32048241	7.58E-05	0.0049991	1
821	Kcnj15	158	0.30944242	7.61E-05	0.00500863	1
1693	Wls	160	-0.3073202	7.72E-05	0.00506479	1
959	Mb	159	0.30819177	7.75E-05	0.00506479	1
830	Kdf1	156	0.31101392	7.76E-05	0.00506479	1
1086	Npr1	159	-0.3079474	7.85E-05	0.00511456	1
137	Antxr1	158	-0.308833	7.88E-05	0.00511709	1
1588	Tmem161b	156	0.31052964	7.97E-05	0.00516394	1
1023	Muc13	156	0.31026691	8.09E-05	0.00522015	1
782	Il4	155	0.3112025	8.10E-05	0.00522015	1
1114	Obox5	152	0.31411236	8.12E-05	0.00522015	1
1712	Ywhag	160	-0.3062119	8.22E-05	0.00526098	1
387	Col9a1	160	-0.3062008	8.22E-05	0.00526098	1
1020	Mtss1	160	-0.3061002	8.27E-05	0.00527742	1
177	Armc4	159	-0.3069592	8.30E-05	0.00527742	1
675	Gpnmb	160	-0.306012	8.31E-05	0.00527742	1

1652	Ung	156	0.30956954	8.41E-05	0.00532572	1
1514	Susd2	153	-0.3123722	8.46E-05	0.00534574	1
533	Epha10	160	-0.3055708	8.52E-05	0.0053698	1
1120	Olfr1387	158	-0.3072889	8.59E-05	0.00540018	1
1620	Tspan1	155	0.310065	8.63E-05	0.00541161	1
1270	Pyurf	146	0.31902287	8.68E-05	0.00543324	1
151	Apoc2	156	0.30882247	8.76E-05	0.00546971	1
1564	Tex22	159	0.30586528	8.83E-05	0.00549541	1
1533	Tal1	160	-0.3048007	8.90E-05	0.00552223	1
996	Mob3b	160	-0.3047656	8.91E-05	0.00552223	1
1669	Vdac3	160	-0.3043782	9.11E-05	0.00562961	1
857	Lao1	152	0.31161437	9.31E-05	0.0057317	1
870	Lgals1	160	-0.30397	9.32E-05	0.0057317	1
90	Actn2	156	-0.3076667	9.34E-05	0.00573273	1
1671	Vmac	158	-0.3052974	9.60E-05	0.0058669	1
578	Fbp2	155	0.30811651	9.61E-05	0.0058669	1
477	Dppa4	156	0.30705677	9.66E-05	0.00588639	1
999	Mpg	156	0.306922	9.73E-05	0.00591362	1
191	Atf5	159	-0.3040618	9.76E-05	0.00591362	1
530	Epgn	160	-0.3031091	9.78E-05	0.00591362	1
758	Hsp90b1	156	-0.3064638	9.98E-05	0.00602411	1
434	Dand5	159	-0.3035104	0.00010063	0.00604617	1
617	Gabrp	160	0.302583	0.00010067	0.00604617	1
1290	Rbm19	129	0.3355203	0.0001015	0.0060734	1
874	Lhx6	153	0.30894202	0.0001021	0.0060734	1
1217	Plxnb3	155	-0.307005	0.00010212	0.0060734	1
716	Hadha	151	-0.310838	0.00010251	0.0060734	1
106	Agtrap	157	-0.3050324	0.00010254	0.0060734	1
1614	Trex2	159	-0.3031675	0.00010256	0.0060734	1
1241	Pramel4	159	-0.3031143	0.00010286	0.00607719	1
1489	Spryd4	155	0.30659321	0.00010445	0.00615657	1
1282	Ralgps2	156	0.30557211	0.00010484	0.0061657	1
7	1700094D03Rik	150	-0.3111973	0.00010611	0.00622555	1
1286	Rax	159	0.30239684	0.00010702	0.0062649	1
1035	Myoz1	149	0.31195283	0.00010755	0.00628117	1
942	Mafg	156	-0.3050648	0.0001078	0.0062814	1
1320	Riok1	155	0.30591439	0.00010839	0.0063016	1
1468	Snx20	158	0.30286793	0.00010973	0.00636468	1
767	Id3	160	-0.300972	0.00011008	0.00637037	1
1653	Usb1	156	0.3045034	0.00011116	0.00641843	1
1582	Tmem123	155	0.30528234	0.00011219	0.00645581	1
1342	Rps21	155	0.30526237	0.00011231	0.00645581	1
1243	Prcp	160	-0.3005458	0.0001127	0.00646306	1
689	Gpt	156	0.30420255	0.000113	0.00646306	1
1147	Pabpc4	156	0.3041699	0.0001132	0.00646306	1

1670	Vipr2	156	0.30404004	0.000114	0.00649444	1
290	Ccnl2	160	-0.3000613	0.00011575	0.00657034	1
1181	Peli1	156	0.30374489	0.00011585	0.00657034	1
18	2310039H08Rik	158	0.30145357	0.00011857	0.00670971	1
888	LOC432463	159	-0.3003916	0.0001195	0.00674729	1
131	Amacr	155	0.30404086	0.00012002	0.00676123	1
198	Atp5a1	160	-0.2988177	0.00012393	0.0069576	1
518	Emc3	156	0.30248724	0.00012405	0.0069576	1
1683	Vwf	159	-0.2996108	0.00012472	0.00697997	1
253	Caap1	158	0.30044696	0.00012527	0.00699542	1
691	Grap2	156	0.30214225	0.00012639	0.00704235	1
313	Cdhr4	159	-0.2993058	0.00012682	0.00705075	1
755	Hsd3b7	155	0.30265791	0.00012933	0.0071749	1
1743	Zfp91	159	-0.2986483	0.00013144	0.00727211	1
99	Adi1	155	0.30232398	0.00013168	0.00727211	1
898	LOC433071	155	-0.3022519	0.00013219	0.00727211	1
1328	Rnd2	160	-0.2976136	0.00013237	0.00727211	1
1695	Wrap73	160	0.29759422	0.00013251	0.00727211	1
686	Gpr82	153	-0.3037931	0.0001348	0.00733263	1
1729	Zfp185	158	-0.2990983	0.0001348	0.00733263	1
525	Enpp6	160	-0.2972596	0.00013495	0.00733263	1
840	Klhl10	159	0.29815892	0.00013499	0.00733263	1
975	Mettl21a	155	0.3018537	0.00013505	0.00733263	1
1155	Papola	160	-0.2970879	0.00013622	0.00737075	1
927	Lrrcc1	156	-0.3007147	0.00013652	0.00737075	1
912	LOC434786	159	-0.2979377	0.00013662	0.00737075	1
1215	Pltp	156	-0.3005855	0.00013747	0.00738912	1
462	Dlx6os1	158	0.29872613	0.00013754	0.00738912	1
465	Dmxl1	159	-0.2976258	0.00013896	0.00743359	1
1301	Rfk	152	0.30417407	0.00013906	0.00743359	1
104	Ago2	157	-0.2994186	0.00013925	0.00743359	1
1718	Zc2hc1b	160	0.29663271	0.00013964	0.00743879	1
447	Dennd4c	158	-0.2983048	0.00014072	0.00748058	1
1112	Oasl2	156	0.30003015	0.00014164	0.00751408	1
357	Clcc1	155	0.30080836	0.00014285	0.00756221	1
335	Cfap43	160	-0.2961084	0.00014367	0.00759023	1
1236	Ppp1r16a	159	-0.2968997	0.00014453	0.00761987	1
497	Efcab12	160	-0.2958842	0.00014543	0.0076514	1
1602	Tnfrsf17	156	0.29945927	0.00014605	0.00766805	1
602	Fpr-rs4	159	-0.2966356	0.00014661	0.00768182	1
724	Hbb-b1	158	-0.2973842	0.00014788	0.00772357	1
934	Lyve1	155	0.30012108	0.00014819	0.00772357	1
837	Klf7	160	-0.2954947	0.00014853	0.00772357	1
1440	Slc5a2	156	0.29913331	0.00014862	0.00772357	1
1471	Snx8	158	0.29712987	0.00014992	0.00775305	1

220	Bcar3	160	-0.2953073	0.00015005	0.00775305	1
1219	Pml	155	0.29988137	0.0001501	0.00775305	1
1303	Rfx1	159	0.29604251	0.00015138	0.00780337	1
979	Mgat1	155	0.29957589	0.00015257	0.0078486	1
176	Armc3	159	-0.2957794	0.00015355	0.00788295	1
1102	Nudt1	157	0.29721452	0.00015676	0.00802152	1
736	Hibadh	156	0.29812248	0.00015688	0.00802152	1
1122	Olfr371	159	-0.2948825	0.00016114	0.00822296	1
599	Foxj1	156	-0.2975365	0.00016186	0.00822569	1
1594	Tmem229b	158	-0.2956583	0.00016225	0.00822569	1
1637	Uaca	158	-0.2956104	0.00016267	0.00822569	1
674	Gpc3	160	0.2938104	0.00016268	0.00822569	1
224	Bcl2l10	157	0.29650593	0.00016281	0.00822569	1
1137	Osbpl7	126	-0.3296751	0.00016347	0.00824271	1
1608	Tox	159	-0.2943789	0.00016555	0.00830495	1
1550	Tcerg1l	160	0.29347922	0.0001656	0.00830495	1
1175	Pdxk	160	-0.2934702	0.00016568	0.00830495	1
1593	Tmem229a	159	-0.2942749	0.00016648	0.00832858	1
1298	Rdh7	152	0.30072272	0.0001669	0.00833332	1
606	Fstl5	159	0.29404519	0.00016854	0.00839765	1
1507	Stx3	158	-0.2949126	0.00016885	0.00839765	1
662	Gm5607	160	0.29304245	0.00016953	0.00841518	1
1531	Sytl5	160	0.29292813	0.00017058	0.00844342	1
507	Eif4a3	160	-0.2929075	0.00017077	0.00844342	1
889	LOC432560	158	0.29450174	0.00017259	0.00851724	1
123	Aldh9a1	155	0.29697824	0.00017512	0.00862535	1
778	Igsf3	160	-0.2922677	0.00017672	0.00864305	1
1563	Tex19.1	155	0.29680517	0.00017673	0.00864305	1
1167	Pde4dip	160	-0.2922571	0.00017682	0.00864305	1
279	Ccdc23	156	0.29586893	0.00017684	0.00864305	1
815	Kcna2	147	-0.3044708	0.00017725	0.00864632	1
306	Cdca7	160	-0.2921175	0.00017815	0.00866601	1
1061	Nefl	160	-0.2920382	0.0001789	0.00866601	1
1592	Tmem219	152	0.29937528	0.00017912	0.00866601	1
164	Arg1	152	0.2993546	0.00017931	0.00866601	1
496	Ecel1	160	0.29199168	0.00017935	0.00866601	1
178	Arr3	155	0.2963525	0.000181	0.00872913	1
1395	Shh	160	0.29178294	0.00018136	0.00873011	1
259	Calcr	160	0.29168987	0.00018227	0.00875421	1
1335	Rpap2	156	0.29526769	0.00018255	0.00875421	1
798	Isl1	155	0.29611106	0.00018332	0.00877439	1
107	Agxt2	159	-0.2922911	0.00018509	0.00884242	1
1203	Pla2g10	149	0.301515	0.00018637	0.00888707	1
1665	Vat1	152	0.29850507	0.00018744	0.00889743	1
1749	Zim1	160	0.29115807	0.00018751	0.00889743	1

1182	Peli3	156	0.2947472	0.00018763	0.00889743	1
54	9030407P20Rik	158	-0.2928189	0.00018874	0.00893333	1
1308	Rgs5	160	-0.2909576	0.00018953	0.0089402	1
783	Il4ra	160	-0.2909514	0.00018959	0.0089402	1
1717	Zbtb4	149	-0.300857	0.00019281	0.00907322	1
922	Lpar1	159	-0.291491	0.00019312	0.00907322	1
212	B630019K06Rik	159	0.2914189	0.00019386	0.00909121	1
273	Ccdc108	156	-0.2940258	0.0001949	0.00909267	1
1391	Sgpl1	156	-0.2940205	0.00019495	0.00909267	1
551	Fabp7	160	-0.290426	0.00019497	0.00909267	1
862	Ldlrad2	158	-0.2921443	0.0001956	0.00909881	1
169	Arhgap5	160	-0.2903445	0.00019581	0.00909881	1
921	Lox	155	0.29481579	0.00019622	0.00910134	1
594	Fibp	152	0.29746557	0.00019786	0.00916044	1
46	5430435G22Rik	156	0.29367162	0.00019856	0.00917622	1
1009	Mrpl4	156	-0.2935193	0.00020015	0.00923312	1
261	Calm1	160	-0.2898541	0.00020098	0.00924744	1
68	Aar2	158	0.29161106	0.00020119	0.00924744	1
1420	Slc25a24	156	0.29328311	0.00020265	0.00928596	1
1081	Nmb	158	0.29142763	0.00020314	0.00928596	1
568	Fam89b	157	0.29228012	0.00020365	0.00928596	1
1317	Rhpn1	159	-0.2904772	0.00020377	0.00928596	1
69	Abca13	159	-0.2904701	0.00020385	0.00928596	1
1083	Nop9	160	0.28953166	0.00020444	0.00929634	1
122	Aldh3b1	158	0.29121368	0.00020545	0.00932464	1
974	Mettl14	152	0.2966914	0.00020596	0.00932464	1
1506	Strbp	158	-0.2911476	0.00020616	0.00932464	1
1488	Sphkap	160	-0.2892702	0.00020729	0.00935911	1
660	Gm5126	159	-0.2900814	0.00020808	0.00937793	1
725	Hbb-b2	158	-0.2907871	0.00021011	0.0094529	1
457	Dip2a	160	-0.2887905	0.00021262	0.00954885	1
398	Cpe	160	-0.2886857	0.0002138	0.00957011	1
759	Htatsf1	158	-0.2904521	0.00021385	0.00957011	1
846	Kmt2a	160	-0.2885512	0.00021533	0.00961938	1
321	Cds1	160	-0.2883582	0.00021753	0.00967998	1
995	Mob3b	155	0.29282411	0.00021773	0.00967998	1
1568	Thbs2	160	-0.2883328	0.00021782	0.00967998	1
1399	Sigirr	158	0.29004909	0.00021842	0.00968956	1
486	Dynlrb2	160	-0.2881599	0.00021982	0.00973465	1
626	Galnt1	153	0.29424952	0.00022259	0.00983533	1
983	Mipol1	156	-0.2914636	0.00022286	0.00983533	1
760	Htr2c	160	-0.2877985	0.00022404	0.00986256	1
439	Dclre1b	156	0.29134411	0.00022425	0.00986256	1
1523	Syp	159	-0.2885103	0.00022601	0.00990968	1
1194	Pidd1	152	-0.2948802	0.00022614	0.00990968	1

764	Hyal1	152	0.29485018	0.00022649	0.00990968	1
392	Coq7	155	0.29194525	0.0002279	0.00994151	1
1674	Vps13b	159	-0.2882971	0.00022856	0.00994151	1
715	Hacl1	156	0.29095734	0.00022881	0.00994151	1
1356	Rxrg	160	-0.2873712	0.00022914	0.00994151	1
60	A630043P06	160	-0.287337	0.00022955	0.00994151	1
356	Clasp1	159	-0.2881872	0.00022988	0.00994151	1
40	4930588G17Rik	159	-0.2881568	0.00023024	0.00994151	1
1382	Serpinb9e	155	0.2917196	0.00023058	0.00994151	1
1104	Nwd1	160	-0.2872389	0.00023073	0.00994151	1
520	Enkur	159	-0.2879166	0.00023316	0.01002105	1
557	Fam187a	159	-0.2878994	0.00023337	0.01002105	1
652	Gm1335	160	-0.2869749	0.00023396	0.01002944	1
71	Abcc4	160	-0.2867242	0.00023705	0.01014511	1
612	G6pc	152	0.29387011	0.00023818	0.01017632	1
351	Ciao1	160	0.28653509	0.00023942	0.01019204	1
1311	Rhebl1	156	0.29007352	0.00023955	0.01019204	1
615	Gabbr2	155	-0.2909657	0.00023975	0.01019204	1
210	B3gnt8	155	0.29084982	0.00024119	0.01023617	1
92	Adamts1	158	0.28806009	0.00024235	0.01026801	1
226	Becn1	157	-0.288861	0.00024348	0.01028731	1
1366	Scamp4	156	0.28974946	0.00024361	0.01028731	1
1753	Zscan26	159	-0.2870118	0.00024445	0.01030579	1
1158	Pcdh8	159	0.28688322	0.0002461	0.01035579	1
1329	Rnf11	160	-0.285982	0.00024645	0.01035579	1
1373	Sectm1a	153	0.29221029	0.00024722	0.0103709	1
784	Impa2	154	0.29095428	0.00025147	0.01053216	1
643	Gja1	160	-0.2853265	0.00025503	0.01064159	1
318	Cdk8	160	-0.285324	0.00025507	0.01064159	1
935	Lyve1	160	-0.2853035	0.00025534	0.01064159	1
522	Enpp2	160	-0.285165	0.00025719	0.01070121	1
1351	Rsu1	158	0.28684909	0.00025808	0.01072073	1
345	Chrna10	155	0.28946419	0.00025904	0.01073052	1
1033	Myl6b	155	0.289455	0.00025916	0.01073052	1
1579	Tmem106b	160	-0.2848846	0.00026098	0.0107882	1
362	Clic1	160	-0.2847254	0.00026315	0.01085381	1
1493	Sri	160	-0.2847059	0.00026342	0.01085381	1
235	Bmp7	160	-0.2846002	0.00026487	0.01089308	1
640	Ggt1	155	0.28898579	0.00026548	0.01089308	1
113	AK129341	156	-0.2880706	0.00026565	0.01089308	1
1446	Slc9a8	158	0.28621696	0.00026667	0.01091704	1
1283	Ran	160	-0.2844301	0.00026722	0.01091704	1
400	Cpt1b	159	0.28526116	0.00026775	0.01091704	1
186	Asns	160	-0.2843777	0.00026795	0.01091704	1
1452	Smg6	156	-0.2874413	0.00027438	0.01110393	1

669	Gnat1	160	-0.2839024	0.00027465	0.01110393	1
991	Mmp3	155	0.28829542	0.00027504	0.01110393	1
1220	Pnoc	156	0.28739457	0.00027504	0.01110393	1
1043	Nans	158	0.28560211	0.00027527	0.01110393	1
1701	Xlr4a	156	-0.2873447	0.00027575	0.01110393	1
1315	Rhox5	158	-0.285552	0.00027599	0.01110393	1
453	Dhcr7	156	0.2872726	0.00027677	0.01110393	1
891	LOC432648	156	-0.2872648	0.00027688	0.01110393	1
1450	Smc5	154	0.28907209	0.0002769	0.01110393	1
1710	Ywhab	158	0.28541029	0.00027801	0.01111445	1
162	Araf	157	0.28629193	0.00027804	0.01111445	1
1459	Snap25	160	-0.2835967	0.00027904	0.01113714	1
820	Kcnip1	160	-0.2835386	0.00027988	0.01115325	1
197	Atp13a5	160	-0.2833343	0.00028286	0.01125428	1
1500	Stard10	160	-0.2832135	0.00028463	0.01130724	1
601	Fpgs	156	0.28647419	0.00028832	0.01143577	1
692	Grin2d	160	-0.2827346	0.00029177	0.01155471	1
1566	Tgfbi	158	-0.2843794	0.00029316	0.01159164	1
1368	Scgb2b27	158	-0.2841621	0.00029645	0.01167253	1
646	Gldc	160	-0.2823741	0.00029725	0.01167253	1
1739	Zfp758	158	0.28409279	0.00029751	0.01167253	1
707	Gtf2h2	152	0.28947767	0.00029778	0.01167253	1
281	Ccdc60	159	-0.2830994	0.00029938	0.01167253	1
1305	Rgcc	159	-0.2830854	0.00029959	0.01167253	1
221	Bcar3	158	-0.2839415	0.00029982	0.01167253	1
754	Hsd3b5	156	0.28565868	0.00030058	0.01167253	1
1703	Xxylt1	158	0.28388855	0.00030064	0.01167253	1
1326	Rln3	160	-0.2821074	0.00030137	0.01167253	1
1560	Tenc1	156	-0.2855889	0.00030165	0.01167253	1
1117	Ogt	160	-0.2820768	0.00030185	0.01167253	1
1143	P2rx3	160	0.28206513	0.00030203	0.01167253	1
1730	Zfp263	156	0.28554296	0.00030236	0.01167253	1
309	Cdh24	156	-0.2855361	0.00030246	0.01167253	1
1094	Nt5dc1	159	0.28289494	0.00030254	0.01167253	1
807	Jade1	152	0.28902567	0.00030464	0.01173498	1
792	Irx1	153	-0.2880767	0.00030508	0.01173498	1
195	Atl3	160	-0.2814222	0.00031219	0.01199045	1
470	Dnajc24	150	0.29029687	0.00031387	0.01203683	1
1183	Penk	159	0.28195153	0.00031754	0.01214331	1
1139	Ostn	160	-0.2810878	0.0003176	0.01214331	1
379	Cntrl	156	-0.2844818	0.00031912	0.0121832	1
985	Mitf	156	-0.2841752	0.00032413	0.01235565	1
1055	Ndrg2	158	0.28230679	0.00032596	0.01240685	1
426	Cyp1b1	155	0.28490348	0.00032681	0.0124207	1
632	Gata2	160	-0.2803464	0.00032991	0.01251987	1

334	Cfap43	159	-0.2811093	0.00033152	0.01252877	1
1429	Slc35a2	160	0.28025084	0.00033153	0.01252877	1
1559	Tekt2	143	0.2959887	0.00033168	0.01252877	1
572	Fasl	152	0.28730319	0.00033215	0.01252877	1
544	Exosc3	155	0.28455537	0.0003326	0.01252877	1
1478	Sox14	160	-0.2801545	0.00033317	0.01253141	1
1273	R3hcc1	158	-0.281787	0.0003347	0.0125705	1
899	LOC433088	160	0.27999432	0.00033591	0.01259733	1
607	Ftcd	156	0.28332235	0.00033843	0.0126731	1
232	Blcap	155	0.28412878	0.00033984	0.0127072	1
1692	Wls	158	-0.281381	0.00034168	0.01275536	1
285	Ccer1	157	-0.2822271	0.00034213	0.01275536	1
637	Gemin4	154	0.28470591	0.00034534	0.01284348	1
1465	Snrnp200	159	-0.2802988	0.0003455	0.01284348	1
1227	Pparg	160	-0.2793979	0.0003463	0.01285453	1
804	Itm2b	158	-0.2810397	0.00034765	0.01288587	1
209	Axin2	160	-0.2792711	0.00034855	0.01290033	1
1600	Tmsb15l	154	-0.2844082	0.00035054	0.01293879	1
1105	Nwd2	159	-0.2800104	0.0003506	0.01293879	1
476	Dpm3	157	0.28169901	0.00035139	0.01294912	1
185	Ascc1	158	-0.2806862	0.00035394	0.01302408	1
1393	Sh3gl2	159	-0.2794487	0.00036075	0.01325562	1
1146	P3h3	159	0.27937694	0.00036207	0.01326927	1
1193	Phlda3	155	-0.2828449	0.00036249	0.01326927	1
1470	Snx31	158	-0.2802038	0.00036269	0.01326927	1
4	1700026L06Rik	159	-0.2792643	0.00036414	0.01329597	1
770	Ifit1	155	0.28273685	0.00036446	0.01329597	1
717	Haus4	157	0.28094518	0.00036502	0.01329738	1
1189	Pgr	158	0.28002141	0.00036605	0.01331569	1
573	Fasn	159	-0.2790909	0.00036735	0.01332598	1
1337	Rpl13	160	-0.2782377	0.00036737	0.01332598	1
208	Axin2	160	-0.2781749	0.00036855	0.01334958	1
772	Ifne	156	-0.2815556	0.00036993	0.0133805	1
51	6820408C15Rik	157	-0.2805318	0.0003727	0.01346156	1
1444	Slc7a11	153	-0.284051	0.00037329	0.01346364	1
272	Ccdc106	151	0.28584818	0.00037383	0.01346405	1
672	Gorasp2	157	-0.280317	0.00037675	0.01354523	1
1555	Tctex1d1	159	-0.2785713	0.00037714	0.01354523	1
679	Gpr137b	158	0.27936873	0.00037831	0.01356784	1
1629	Ttll8	159	-0.27843	0.00037985	0.01360348	1
1641	Ube2q2	155	0.28188314	0.00038037	0.01360348	1
1154	Panx3	156	0.28084963	0.00038326	0.01368771	1
1654	Usp16	160	-0.2773045	0.00038518	0.01370428	1
1617	Trim8	156	-0.280739	0.00038539	0.01370428	1
135	Ankrd54	160	-0.277285	0.00038556	0.01370428	1

53	8430408G22Rik	156	0.28068676	0.0003864	0.01370428	1
1662	Uty	151	-0.2851775	0.00038642	0.01370428	1
109	AI987944	159	-0.2780187	0.00038782	0.0137154	1
740	Hist1h4i	158	-0.2788647	0.00038803	0.0137154	1
231	Birc5	153	0.28324494	0.00038853	0.0137154	1
1297	Rdh14	160	-0.277102	0.00038915	0.0137154	1
687	Gpr83	156	0.28053097	0.00038942	0.0137154	1
869	Letmd1	158	0.27872666	0.00039074	0.01374267	1
1369	Scml1	158	-0.2785904	0.00039342	0.01380471	1
1469	Snx27	159	-0.2777069	0.00039397	0.01380471	1
1520	Syn3	160	-0.2768507	0.00039413	0.01380471	1
1204	Pla2g7	146	-0.2892313	0.000399	0.0139564	1
196	Atn1	158	-0.2782613	0.00039998	0.01395666	1
960	Mbd2	156	-0.2799723	0.00040044	0.01395666	1
1636	Tyms	158	0.27822765	0.00040066	0.01395666	1
944	Magee1	159	-0.2773375	0.00040136	0.01396222	1
1750	Zmiz1	158	-0.2781319	0.00040259	0.01397631	1
115	Akna	158	-0.2780887	0.00040346	0.01397631	1
933	Ly6g5b	160	-0.2763714	0.00040378	0.01397631	1
738	Hist1h2bc	160	-0.2763624	0.00040396	0.01397631	1
384	Col4a5	160	-0.2762942	0.00040536	0.01399097	1
730	Hectd2	160	-0.2762878	0.00040549	0.01399097	1
581	Fbxo2	160	-0.2761871	0.00040755	0.0140432	1
314	Cdhr4	151	-0.2840433	0.0004086	0.01405639	1
563	Fam25c	156	-0.2795466	0.00040904	0.01405639	1
727	Hc	158	-0.2777657	0.00041005	0.01406567	1
6	1700052N19Rik	155	0.28032284	0.00041111	0.01406567	1
986	Mknk1	159	0.27685793	0.00041116	0.01406567	1
85	Acot2	158	-0.2776942	0.00041152	0.01406567	1
288	Ccnd3	155	-0.2801454	0.00041475	0.01414783	1
575	Fau	160	-0.2758125	0.00041532	0.01414783	1
39	4930548F15Rik	159	-0.2766446	0.00041559	0.01414783	1
1545	Tbrg4	155	0.28003808	0.00041697	0.01417579	1
875	Lhx8	160	0.2757028	0.00041762	0.0141789	1
1152	Palm2	156	-0.2788868	0.00042269	0.01433222	1
1659	Utp14b	160	-0.2753528	0.00042503	0.01438559	1
956	Masp2	156	0.27875853	0.0004254	0.01438559	1
207	Avp	160	0.27523461	0.00042756	0.01442469	1
440	Dctn1	156	-0.2786216	0.0004283	0.01442469	1
1657	Usp31	158	-0.2768943	0.00042832	0.01442469	1
139	Anxa2	160	-0.2751684	0.00042899	0.01442469	1
1495	Sry	156	-0.2785706	0.00042939	0.01442469	1
1741	Zfp790	160	-0.2751118	0.00043021	0.01443328	1
166	Arhgap22	157	0.27754451	0.0004328	0.01450106	1
1213	Plp2	158	0.27657797	0.00043514	0.0145484	1

433	Dalrd3	156	-0.2782925	0.00043536	0.0145484	1
1302	Rfx1	160	-0.2748253	0.00043644	0.01456552	1
1673	Vps11	155	0.2790748	0.00043734	0.0145733	1
140	Anxa2	157	-0.2772701	0.00043875	0.0145733	1
280	Ccdc58	159	-0.2755217	0.00043964	0.0145733	1
1694	Wnt1	160	-0.2746775	0.00043969	0.0145733	1
389	Commd7	160	-0.2746663	0.00043993	0.0145733	1
330	Cep112	160	-0.2746584	0.00044011	0.0145733	1
1044	Nap1l3	159	-0.2754425	0.00044138	0.01459025	1
239	Bpifb1	160	0.27456863	0.00044209	0.01459025	1
230	Bik	158	0.27624874	0.00044234	0.01459025	1
971	Meis1	136	-0.2970658	0.00044474	0.01465069	1
1686	Wdr63	158	-0.2760351	0.00044707	0.01470819	1
395	Cox7b	160	-0.2742569	0.00044905	0.01475422	1
1118	Olfm1	160	-0.2742153	0.00044998	0.01476038	1
1422	Slc25a41	158	-0.2758611	0.00045096	0.01476038	1
636	Gclc	155	0.27845346	0.00045097	0.01476038	1
668	Gnao1	160	-0.2740196	0.00045441	0.01485379	1
456	Dido1	156	-0.2773751	0.00045559	0.01487319	1
699	Grm8	160	-0.2738695	0.00045783	0.01492733	1
683	Gpr165	159	0.2746577	0.00045901	0.0149466	1
591	Fgd6	155	0.27802349	0.00046063	0.01498026	1
929	Lsm4	155	0.27775033	0.00046687	0.01516368	1
492	Ear6	159	-0.2742751	0.00046784	0.01517581	1
624	Gal3st2	160	-0.2732903	0.00047126	0.01526749	1
828	Kctd8	160	-0.2732365	0.00047253	0.01527508	1
1462	Snapc3	159	-0.2740674	0.0004727	0.01527508	1
887	LOC432456	160	-0.2731815	0.00047383	0.01529022	1
1017	Mthfd2	159	0.27399643	0.00047437	0.01529022	1
739	Hist1h2bc	146	0.2854659	0.00047832	0.01539806	1
111	Aip	156	-0.2763617	0.00047895	0.01539886	1
1184	Pex13	160	-0.2729048	0.0004804	0.01542623	1
814	Kcmf1	159	-0.2736679	0.00048217	0.01546351	1
77	Acads	155	0.27692783	0.00048612	0.01557042	1
293	Cct2	160	-0.2725531	0.00048889	0.01563943	1
23	2610020H08Rik	154	0.27758054	0.00049146	0.01570204	1
906	LOC433740	155	-0.276625	0.00049339	0.01572481	1
1125	Olfr561	158	-0.2740421	0.00049352	0.01572481	1
1436	Slc48a1	159	-0.2731782	0.00049402	0.01572481	1
1480	Spa17	160	-0.2722721	0.00049576	0.01576038	1
1590	Tmem203	130	0.30116148	0.00049805	0.01581342	1
147	Aph1b	154	-0.2770749	0.00050374	0.01597423	1
1676	Vps51	159	-0.2727117	0.00050557	0.01601212	1
1192	Phgdh	156	-0.2750806	0.00051006	0.01613425	1
1267	Ptpdc1	159	-0.2724017	0.00051337	0.01621904	1

1473	Soga3	160	-0.2715136	0.00051477	0.01624299	1
850	Krt32	159	-0.2722993	0.00051598	0.0162609	1
535	Eqtn	160	-0.2714297	0.00051691	0.01627031	1
1211	Plekhm2	157	-0.2739156	0.00051783	0.01627895	1
1744	Zfp952	156	-0.2746918	0.00051986	0.01630356	1
1288	Rbl2	159	-0.2721146	0.0005207	0.01630356	1
1490	Sptb	159	-0.2721049	0.00052095	0.01630356	1
467	Dnah3	158	-0.2729365	0.00052117	0.01630356	1
1157	Pax3	158	-0.2728116	0.00052438	0.016381	1
485	Dus2	159	-0.2719506	0.00052493	0.016381	1
28	2900092C05Rik	160	-0.2710672	0.00052627	0.01640254	1
684	Gpr165	160	0.27102891	0.00052726	0.01641353	1
1517	Svs3a	159	0.27172641	0.00053077	0.01648384	1
510	Eif5	160	-0.270893	0.00053082	0.01648384	1
1030	Mx1	155	0.27497982	0.00053467	0.01658313	1
1331	Rnf138rt1	147	0.28188299	0.00054241	0.0167972	1
1295	Rcc1	154	0.27553598	0.00054289	0.0167972	1
1163	Pcsk1n	159	0.27115442	0.00054592	0.01687058	1
604	Frmd5	159	-0.2711168	0.00054693	0.01688131	1
1542	Tbc1d9	157	-0.2726799	0.00055013	0.01693362	1
381	Cog2	155	0.27437541	0.00055062	0.01693362	1
745	Hnrnpk	138	-0.2903737	0.00055062	0.01693362	1
189	Atf2	148	-0.2805731	0.00055229	0.01696441	1
141	Aox1	152	0.27687699	0.00055408	0.01699884	1
188	Aspn	155	0.27411276	0.00055768	0.01706181	1
1170	Pdgfrb	160	-0.2698757	0.00055813	0.01706181	1
757	Hsp90aa1	147	-0.2812805	0.00055814	0.01706181	1
1702	Xpo1	136	0.29204126	0.00056091	0.01712595	1
1164	Pdcd2	158	-0.2713781	0.00056258	0.0171498	1
1621	Tspan15	160	-0.2696977	0.00056304	0.0171498	1
62	A830029E22Rik	159	-0.2704267	0.00056578	0.01721274	1
628	Galnt13	160	-0.2695325	0.00056763	0.01724849	1
1643	Ublcp1	156	0.27284804	0.00056877	0.01726237	1
1370	Scp2	153	0.27541613	0.00056967	0.01726513	1
1748	Zg16	156	0.27278709	0.00057046	0.01726513	1
205	Atp6v1c2	155	0.27362988	0.00057089	0.01726513	1
987	Mllt1	137	-0.2904941	0.00057411	0.0173418	1
811	Jrkl	158	-0.270937	0.00057484	0.0173433	1
262	Capza1	155	0.27341197	0.00057695	0.01738631	1
248	C2cd5	159	-0.2698839	0.00058103	0.01747316	1
1071	Ngfr	160	0.26905146	0.0005812	0.01747316	1
1277	Rab39	156	0.27217089	0.00058777	0.01764977	1
977	Mfsd6l	153	0.27464781	0.00059112	0.01771576	1
992	Mmp7	148	0.2791168	0.0005918	0.01771576	1
910	LOC434271	159	-0.2694993	0.00059206	0.01771576	1

1018	Mthfsd	146	-0.2808921	0.00059416	0.01775776	1
1751	Zpld1	158	-0.2702228	0.00059522	0.01776855	1
504	Eif2ak3	160	-0.2683493	0.00060155	0.01793659	1
998	Morc3	160	-0.2682505	0.00060446	0.0180024	1
1352	Rtf1	158	-0.2697206	0.00060994	0.0181442	1
89	Acta2	158	-0.2696845	0.00061101	0.01815491	1
1498	St8sia4	150	0.27658738	0.00061199	0.01816298	1
1453	Smim11	158	0.26959837	0.00061357	0.01817615	1
1530	Sytl5	160	0.26793481	0.00061387	0.01817615	1
1441	Slc6a17	157	-0.2703779	0.00061533	0.0181982	1
1327	Rnasel	159	0.26853139	0.00062067	0.01830528	1
371	Cmtr1	153	0.27362069	0.00062095	0.01830528	1
1371	Sdr16c5	156	-0.2710304	0.0006211	0.01830528	1
1733	Zfp384	158	-0.2691402	0.00062737	0.0184686	1
1364	Sbno2	151	0.27508356	0.00062993	0.0185227	1
789	Iqsec1	160	-0.2673219	0.00063251	0.01856237	1
296	Cd247	154	0.27235844	0.00063274	0.01856237	1
228	Bet1	152	0.27398724	0.00063628	0.01863881	1
1045	Napb	156	-0.2705124	0.00063681	0.01863881	1
565	Fam58b	138	0.28716147	0.00063802	0.01865267	1
1411	Slc16a5	156	-0.270398	0.00064033	0.0186931	1
702	Gsdmcl-ps	158	-0.2686829	0.00064143	0.0186931	1
1726	Zdhhc9	160	-0.2670288	0.0006416	0.0186931	1
96	Adat1	156	0.27030035	0.00064335	0.01872247	1
777	Ighg2a	159	-0.2676811	0.00064686	0.01880319	1
708	Gucd1	147	0.27810144	0.00064832	0.01882002	1
167	Arhgap29	160	-0.2667959	0.00064892	0.01882002	1
1165	Pdcd6ip	159	-0.2674909	0.00065286	0.01891275	1
294	Cct2	160	-0.2665972	0.00065522	0.01894411	1
1574	Tlr6	158	0.26823665	0.00065543	0.01894411	1
1087	Nptx2	159	-0.2673352	0.0006578	0.01899121	1
1607	Tor4a	155	0.27063278	0.00065955	0.01902027	1
579	Fbrs	160	-0.2664146	0.00066105	0.01904194	1
649	Gm10549	160	0.26637012	0.00066248	0.0190616	1
52	6820408C15Rik	160	-0.2662862	0.00066519	0.01910179	1
936	Lyz2	158	-0.2679245	0.00066538	0.01910179	1
947	Maoa	160	-0.2662374	0.00066677	0.01912005	1
419	Cubn	160	-0.2662062	0.00066778	0.01912743	1
877	Lig3	158	-0.2677399	0.00067134	0.01917292	1
338	Cfp	157	-0.2685477	0.00067215	0.01917292	1
980	Mia	159	-0.2668786	0.00067251	0.01917292	1
546	Eya1	160	-0.2660261	0.00067364	0.01917292	1
391	Copz2	156	-0.2693417	0.00067367	0.01917292	1
1385	Sf3b6	126	0.29891822	0.00067388	0.01917292	1
373	Cngb1	155	0.27009731	0.00067667	0.01921167	1

1557	Tdrd3	156	0.26924634	0.00067675	0.01921167	1
1435	Slc41a3	160	-0.2659055	0.00067759	0.01921398	1
1738	Zfp748	155	0.26994203	0.00068171	0.0193093	1
816	Kcna5	158	0.26730972	0.0006854	0.01939234	1
661	Gm520	157	0.26804049	0.00068874	0.01946508	1
1389	Sfxn4	159	-0.2662508	0.00069321	0.01955122	1
531	Epha1	158	-0.2670492	0.00069405	0.01955122	1
34	4921531P07Rik	156	-0.2687181	0.00069409	0.01955122	1
1293	Rc3h1	159	-0.2661264	0.00069739	0.01962237	1
867	Lenep	158	0.26688694	0.00069949	0.01963062	1
305	Cdc5l	159	-0.2660477	0.00070004	0.01963062	1
1427	Slc2a1	160	-0.2652248	0.00070029	0.01963062	1
1210	Plekhj1	158	0.26684915	0.00070076	0.01963062	1
375	Cnnm2	160	-0.2651236	0.00070373	0.019692	1
592	Fgf12	160	-0.2650666	0.00070567	0.0197247	1
211	B3gnt9	158	-0.2666052	0.00070903	0.01978307	1
901	LOC433588	159	-0.2657746	0.00070931	0.01978307	1
128	Alox12e	157	0.26736162	0.00071152	0.01982303	1
1421	Slc25a32	155	0.26895217	0.00071465	0.01988399	1
574	Fastkd5	155	0.26890242	0.00071635	0.01988399	1
300	Cd96	156	0.26802975	0.0007173	0.01988399	1
1510	Suco	160	-0.2647282	0.0007173	0.01988399	1
1727	Zfand2a	152	0.27144844	0.00071762	0.01988399	1
1110	Oas1b	154	0.26960584	0.00072145	0.0199684	1
1350	Rsad2	154	-0.2695727	0.00072258	0.01996867	1
168	Arhgap4	156	0.2678473	0.00072357	0.01996867	1
1226	Postn	156	-0.2678353	0.00072398	0.01996867	1
1232	Ppm1a	156	-0.2678175	0.00072459	0.01996867	1
1006	Mrgprb4	160	-0.2644947	0.00072542	0.01996994	1
555	Fam169b	155	0.26856385	0.00072797	0.02001691	1
80	Accs	158	0.2660347	0.0007287	0.02001691	1
49	6030419C18Rik	156	-0.2676422	0.00073068	0.02004947	1
57	9330109E03Rik	158	-0.2658351	0.0007357	0.02015686	1
2	1600029I14Rik	159	-0.2649921	0.00073652	0.02015686	1
1123	Olfr424	158	-0.2657916	0.00073724	0.02015686	1
1658	Usp54	159	-0.2649377	0.00073844	0.02015686	1
928	Lsm1	156	-0.2674172	0.00073855	0.02015686	1
836	Kifc2	160	-0.2640379	0.00074156	0.02018885	1
1431	Slc38a1	158	0.26566576	0.00074169	0.02018885	1
173	Arl10	160	0.26401935	0.00074223	0.02018885	1
908	LOC433773	158	-0.2656321	0.00074289	0.02018885	1
542	Etfdh	159	0.26477451	0.00074425	0.02020427	1
690	Gpx6	158	0.26555222	0.00074573	0.02022295	1
881	LOC195357	158	0.26552293	0.00074678	0.02022976	1
1134	Orc5	158	0.26539781	0.00075126	0.02030987	1

1212	Pllp	160	-0.2637659	0.00075133	0.02030987	1
82	Acer2	158	-0.2652332	0.0007572	0.0204467	1
180	Arrdc3	158	-0.2651652	0.00075966	0.02049145	1
1534	Tal1	160	-0.2634747	0.00076192	0.02053072	1
808	Jakmip3	158	-0.265017	0.00076505	0.02057624	1
445	Ddx52	159	0.26419465	0.00076523	0.02057624	1
966	Mdm1	156	-0.2666328	0.0007666	0.02059145	1
243	Btg3	156	0.26651093	0.00077105	0.02068904	1
1503	Stau1	152	0.26972197	0.00077828	0.02086112	1
559	Fam20a	160	-0.2629238	0.00078233	0.02094762	1
1224	Pomc	160	0.26281756	0.00078632	0.02103154	1
1047	Narfl	156	0.26607577	0.00078711	0.02103154	1
256	Cadm3	160	-0.2625691	0.00079573	0.02123951	1
488	E230008N13Rik	158	-0.2640204	0.00080226	0.0213682	1
429	Cypt1	159	-0.2631903	0.00080285	0.0213682	1
83	Acer3	160	0.26237717	0.00080307	0.0213682	1
1645	Ubxn10	158	-0.2639303	0.00080571	0.02141594	1
404	Cradd	159	-0.2630723	0.00080738	0.02143799	1
163	Arf6	155	0.26630458	0.0008101	0.02148491	1
1197	Pik3cb	157	-0.2646186	0.00081083	0.02148491	1
1042	Nanp	156	0.26541272	0.00081219	0.02148644	1
1569	Ticam2	158	-0.2637516	0.00081258	0.02148644	1
879	Lmbr1l	156	0.26535181	0.00081453	0.02151572	1
1546	Tbx2	158	0.26344447	0.00082451	0.02175687	1
756	Hsf2	152	0.26837862	0.0008287	0.0218447	1
317	Cdk5rap2	159	-0.2625004	0.00082966	0.02184746	1
237	Bpgm	160	-0.2616626	0.00083095	0.02185877	1
165	Arhgap18	158	0.26312977	0.00083691	0.02199285	1
1244	Prdm2	136	-0.2831158	0.0008384	0.02200922	1
93	Adamts18	152	0.2680596	0.00084111	0.02204388	1
1497	St3gal6	160	-0.2613992	0.00084145	0.02204388	1
1049	Natd1	148	0.27147038	0.00084542	0.02212515	1
1387	Sft2d2	157	0.26364433	0.00084907	0.02219773	1
825	Kcnq1ot1	159	-0.2619444	0.00085187	0.02220363	1
577	Fbp1	154	0.2660745	0.00085199	0.02220363	1
1706	Yipf5	158	0.26273831	0.00085257	0.02220363	1
1294	Rcbtb2	152	0.26776337	0.00085278	0.02220363	1
460	Dlx2	154	0.26581372	0.00086244	0.02243226	1
1687	Wdr77	159	-0.2615269	0.00086891	0.02255743	1
411	Csdc2	158	-0.2623341	0.00086902	0.02255743	1
406	Creb3l3	155	0.26477625	0.0008704	0.02257004	1
1685	Wdpcp	157	0.26308637	0.0008717	0.02258084	1
222	Bche	160	-0.2606091	0.00087368	0.02258708	1
1074	Nhsl1	160	-0.2606082	0.00087371	0.02258708	1
732	Herpud1	158	-0.2621531	0.00087649	0.02261645	1

361	Clec4a3	159	-0.2613125	0.00087778	0.02261645	1
17	2310035C23Rik	158	-0.2621102	0.00087826	0.02261645	1
320	Cds1	158	-0.2621068	0.0008784	0.02261645	1
352	Cidea	160	-0.2604101	0.00088197	0.02268536	1
1731	Zfp353-ps	158	-0.2619847	0.00088348	0.02269142	1
1361	Samd14	160	-0.260362	0.00088399	0.02269142	1
41	4931408C20Rik	160	-0.2603102	0.00088616	0.02272436	1
1377	Serpina1a	152	0.26685991	0.0008893	0.02276963	1
590	Fezf1	160	0.2602258	0.00088972	0.02276963	1
1075	Ninj1	160	-0.2601335	0.00089362	0.0228352	1
658	Gm471	158	-0.2617319	0.00089407	0.0228352	1
909	LOC433775	159	-0.2607116	0.00090308	0.02304173	1
926	Lrrc40	153	0.26559803	0.00090624	0.02304173	1
407	Crh	157	0.26225031	0.00090665	0.02304173	1
274	Ccdc109b	160	-0.2598155	0.00090719	0.02304173	1
952	Mapk8	153	-0.2655701	0.00090741	0.02304173	1
1651	Unc80	159	-0.2606061	0.00090759	0.02304173	1
1115	Obox6	155	0.26383832	0.00090941	0.02306499	1
1585	Tmem144	152	0.26630982	0.00091224	0.02311373	1
1540	Tasp1	152	0.26618573	0.00091749	0.0232236	1
267	Cbln2	146	-0.2714452	0.00091924	0.02324486	1
883	LOC240906	158	-0.2611132	0.0009205	0.02325055	1
1322	Ripk3	156	0.26273059	0.00092129	0.02325055	1
1284	Ranbp2	157	-0.261856	0.00092358	0.02328523	1
1259	Psmc4	149	-0.2686054	0.00092559	0.0233127	1
1538	Tars	158	0.26092915	0.0009285	0.02336301	1
964	Mccc2	155	0.2631937	0.00093715	0.02353608	1
856	Lamp1	157	-0.2615334	0.00093765	0.02353608	1
621	Gak	157	-0.2615088	0.00093873	0.02353608	1
1418	Slc22a8	160	-0.2590849	0.00093908	0.02353608	1
1401	Sin3b	159	-0.2597811	0.00094358	0.02362572	1
623	Gal	160	0.25891193	0.00094677	0.0236757	1
344	Chia1	152	0.26547517	0.00094808	0.0236757	1
3	1700019L03Rik	159	-0.2596736	0.00094837	0.0236757	1
611	G3bp1	156	0.26205335	0.00095089	0.02371546	1
149	Apoa1bp	159	-0.259557	0.00095358	0.02375933	1
536	Erlin1	155	0.26274029	0.00095712	0.02381949	1
554	Fam151b	160	-0.2586649	0.00095787	0.02381949	1
304	Cdc42ep2	155	0.26264558	0.00096134	0.02388256	1
805	Itpk1	160	-0.2584551	0.00096739	0.02400927	1
1079	Nkx1-1	156	-0.2616624	0.00096837	0.02401038	1
1238	Ppp2r3d	160	-0.2583457	0.00097238	0.02408634	1
676	Gpr101	160	0.25829994	0.00097448	0.02411482	1
1057	Ndufs1	160	-0.2581608	0.00098088	0.02424968	1
1177	Pef1	144	-0.2717494	0.00098491	0.02429596	1

1587	Tmem160	159	-0.2588376	0.00098635	0.02429596	1
665	Gm816	152	-0.2646118	0.00098651	0.02429596	1
831	Khk	154	0.26292034	0.00098657	0.02429596	1
650	Gm1140	156	-0.2611461	0.00099191	0.02440221	1
1483	Spata18	159	-0.2586712	0.00099407	0.02440221	1
1722	Zcchc7	159	-0.2586585	0.00099466	0.02440221	1
409	Crnde	158	-0.2594591	0.00099472	0.02440221	1
201	Atp5o	160	-0.2578339	0.00099607	0.02441196	1
1056	Ndrg3	160	-0.2577589	0.00099959	0.02447455	1
55	9130024F11Rik	160	-0.257657	0.00100438	0.02456835	1
479	Drd3	160	0.25762787	0.00100576	0.0245784	1
102	Adrb2	155	0.26156792	0.00101059	0.02466839	1
1107	Nxn	160	-0.2575092	0.00101138	0.02466839	1
957	Matn1	152	0.26403046	0.00101318	0.02468868	1
1547	Tbx22	159	-0.2580713	0.00102238	0.02488234	1
1048	Nat2	155	0.26128256	0.00102401	0.02488234	1
1376	Serpina10	152	0.26378294	0.00102474	0.02488234	1
682	Gpr156	160	-0.2572047	0.00102593	0.02488234	1
1089	Nr4a2	159	0.25798901	0.00102632	0.02488234	1
539	Esr1	160	0.25718253	0.00102699	0.02488234	1
22	2610008E11Rik	159	-0.2578209	0.00103441	0.02502811	1
1638	Uap1l1	158	-0.2586	0.00103539	0.02502811	1
432	Dab2	139	-0.2753424	0.00103596	0.02502811	1
1723	Zcchc8	158	0.2585031	0.00104007	0.02510368	1
1174	Pdlim4	159	-0.2574905	0.00105049	0.02533113	1
785	Ina	152	-0.2631296	0.00105582	0.02543569	1
1145	P2ry2	160	-0.2564603	0.0010623	0.02555402	1
826	Kctd10	155	-0.2604585	0.00106369	0.02555402	1
1160	Pcdhb20	160	-0.2564312	0.00106375	0.02555402	1
12	1810065E05Rik	156	0.25959955	0.00106559	0.02557405	1
1624	Ttc16	150	-0.2644951	0.00107267	0.02571973	1
586	Fbxw17	155	0.26014767	0.00107902	0.02584772	1
1755	Zufsp	156	-0.2592513	0.00108285	0.02591517	1
1101	Nudcd2	153	0.26159265	0.0010898	0.02605705	1
937	Mab21l1	160	-0.2558292	0.00109406	0.02612631	1
413	Cstf2	160	-0.2557625	0.00109747	0.02612631	1
1343	Rps4x	159	-0.2565371	0.00109817	0.02612631	1
1496	Ssna1	158	-0.2573239	0.00109862	0.02612631	1
1584	Tmem132a	159	-0.2565212	0.00109898	0.02612631	1
1034	Myo7a	158	-0.2573136	0.00109915	0.02612631	1
670	Gnb3	158	0.25727344	0.0011012	0.02612631	1
1095	Nt5dc2	156	0.25886727	0.00110219	0.02612631	1
483	Dtd1	160	-0.2556663	0.0011024	0.02612631	1
1266	Ptn	160	-0.2556554	0.00110296	0.02612631	1
21	2610005L07Rik	158	-0.2571809	0.00110592	0.02617215	1

370	Cml3	155	-0.2594859	0.00111233	0.02629937	1
526	Entpd1	155	0.25945549	0.00111388	0.02631165	1
1063	Nek2	160	-0.2553673	0.00111786	0.02638098	1
1195	Pifo	159	-0.2561084	0.00112025	0.02641302	1
1255	Psap	160	-0.2552914	0.00112181	0.02642147	1
402	Cr1l	158	-0.256856	0.00112268	0.02642147	1
1096	Ntrk1	159	0.25601201	0.00112527	0.02643841	1
1554	Tcta	156	0.25841288	0.00112548	0.02643841	1
1344	Rps5	160	0.25512847	0.00113034	0.0265059	1
1604	Tnk1	155	0.25913384	0.00113044	0.0265059	1
1064	Nek5	155	0.25907425	0.00113353	0.02655392	1
765	Hyou1	158	-0.2566009	0.001136	0.02658563	1
1586	Tmem14c	154	0.25981904	0.00113764	0.02658563	1
1126	Olfr646	158	-0.2565626	0.00113801	0.02658563	1
1	1110032A03Rik	151	0.26228104	0.00114061	0.02662197	1
1196	Pigf	155	0.25889355	0.00114295	0.026652	1
245	C130036L24Rik	158	0.25644167	0.00114439	0.02666119	1
136	Ankrd6	160	-0.2548278	0.00114625	0.02668015	1
298	Cd5	155	0.25878914	0.00114842	0.02669378	1
324	Cela1	155	0.25876534	0.00114967	0.02669378	1
1332	Rnf170	156	0.25794409	0.00114998	0.02669378	1
446	Ddx59	159	-0.2555058	0.00115198	0.02671581	1
1059	Necab3	158	-0.2562287	0.0011557	0.0267548	1
98	Adh7	156	0.25783481	0.00115576	0.0267548	1
441	Dcxr	155	0.25857488	0.00115973	0.02678199	1
706	Gtf2h1	156	0.25775709	0.00115989	0.02678199	1
154	Appl2	160	-0.2545692	0.00116009	0.02678199	1
749	Hoxd9	160	-0.2545133	0.0011631	0.02682345	1
1109	Nxph1	160	-0.2544968	0.00116399	0.02682345	1
1477	Sorl1	160	-0.2544212	0.00116808	0.02689325	1
776	Igfbp5	160	-0.2543544	0.0011717	0.02695233	1
677	Gpr108	160	-0.2543033	0.00117448	0.02697183	1
35	4921531P07Rik	160	-0.2542998	0.00117467	0.02697183	1
1720	Zcchc17	159	-0.2550205	0.00117812	0.0270127	1
529	Epb4.1l5	154	0.2590439	0.00117857	0.0270127	1
915	LOC545466	158	0.25575041	0.00118146	0.02705452	1
1525	Syt1	160	-0.2540748	0.00118698	0.02715646	1
270	Cbx2	156	0.25713828	0.00119323	0.02727516	1
1091	Nrn1	160	-0.2538808	0.00119768	0.02735229	1
1595	Tmem248	155	0.25767636	0.00120829	0.02756975	1
558	Fam206a	158	0.25522447	0.00121039	0.02759307	1
1347	Rpsa	159	-0.2543436	0.00121549	0.02766685	1
181	Arsg	156	-0.2567284	0.0012158	0.02766685	1
1475	Sorbs3	160	-0.2534922	0.00121939	0.02770543	1
896	LOC432906	159	-0.2542527	0.0012206	0.02770543	1

1375	5-Sep	72	0.37375617	0.00122076	0.02770543	1
835	Kif9	158	-0.254965	0.0012249	0.02777469	1
900	LOC433525	160	-0.2533364	0.0012282	0.02782465	1
129	Alpk2	160	-0.2533125	0.00122956	0.0278306	1
255	Cacnb2	158	-0.2548262	0.00123274	0.02787781	1
1263	Pthlh	158	-0.2547309	0.00123814	0.02797508	1
79	Acbd3	158	-0.2546844	0.00124078	0.02799083	1
385	Col5a3	160	-0.2531112	0.00124103	0.02799083	1
43	4933434E20Rik	158	-0.2546189	0.00124451	0.02801899	1
741	Hmgcs2	154	0.25782936	0.00124542	0.02801899	1
74	Ablim1	158	-0.2546002	0.00124558	0.02801899	1
1562	Tex15	158	-0.2545789	0.0012468	0.0280216	1
1754	Zscan4a	152	0.25942639	0.00124894	0.02802282	1
292	Ccser2	158	-0.2544818	0.00125236	0.02802282	1
1338	Rplp0	160	-0.2529087	0.00125267	0.02802282	1
1682	Vwa5a	155	0.25688299	0.0012527	0.02802282	1
455	Dhx30	154	0.25769378	0.00125309	0.02802282	1
763	Htra2	158	-0.2544627	0.00125346	0.02802282	1
633	Gata3	160	-0.252816	0.00125803	0.02808169	1
1065	Nek7	156	-0.2559752	0.00125829	0.02808169	1
801	Itga8	160	-0.2527834	0.00125992	0.02809345	1
1359	S100b	160	-0.2526594	0.00126714	0.0282296	1
597	Folr2	158	0.25420211	0.00126851	0.02823535	1
376	Cnst	153	-0.2582272	0.00126972	0.02823767	1
1688	Wdtc1	156	-0.2557423	0.00127171	0.0282572	1
1396	Shh	156	0.25566456	0.00127621	0.02833257	1
1424	Slc27a3	159	-0.2532605	0.00127756	0.02833773	1
1247	Prkcq	159	-0.2532184	0.00128003	0.02836785	1
312	Cdh7	158	-0.2539541	0.00128298	0.02840863	1
1349	Rrp12	158	0.25393312	0.00128421	0.02841114	1
1615	Trh	155	0.25630703	0.00128587	0.02842306	1
1222	Polk	158	-0.2538232	0.00129069	0.02850479	1
1027	Mup5	160	-0.2521994	0.00129424	0.02855849	1
882	LOC231914	155	0.2561129	0.00129723	0.02859969	1
1253	Prpsap2	160	0.25209806	0.00130028	0.02862985	1
1188	Pgm3	158	-0.2536516	0.00130085	0.02862985	1
1272	Qsox1	158	-0.2535979	0.00130404	0.02867537	1
170	Arhgef2	160	-0.2519784	0.00130745	0.02872556	1
1642	Ube2t	160	0.25175892	0.00132069	0.02896547	1
343	Cherp	158	-0.2533199	0.00132069	0.02896547	1
1000	Mpi	159	-0.2525177	0.00132178	0.02896547	1
415	Ctf1	158	0.25327602	0.00132334	0.02897458	1
1567	Tgm3	158	-0.2532153	0.00132701	0.02901519	1
569	Fanci	159	-0.2523966	0.00132913	0.02901519	1
1200	Pitx1	159	-0.2523931	0.00132933	0.02901519	1

576	Fbln1	160	-0.2516098	0.00132975	0.02901519	1
332	Cep57	158	-0.2531377	0.00133171	0.02903304	1
1406	Slc15a1	147	-0.2621878	0.00133646	0.02911183	1
1348	Rrm1	159	-0.252211	0.00134044	0.02917357	1
1591	Tmem216	158	-0.2529224	0.00134484	0.02924419	1
1233	Ppm1f	147	-0.2619561	0.00135018	0.02933531	1
1660	Utp3	159	-0.2519322	0.00135762	0.02945179	1
1084	Npdc1	160	-0.2511339	0.00135906	0.02945179	1
911	LOC434781	157	-0.2534764	0.00135934	0.02945179	1
1191	Phf20l1	147	-0.2617761	0.00136093	0.02945179	1
73	Abl1	159	-0.2518725	0.00136132	0.02945179	1
342	Chd2	160	-0.2509992	0.00136747	0.0295454	1
895	LOC432871	149	-0.25992	0.00136851	0.0295454	1
1521	Syne1	160	-0.2509551	0.00137022	0.0295454	1
1709	Ywhab	160	-0.250954	0.00137029	0.0295454	1
919	LOC546041	159	-0.2515637	0.00138063	0.0297432	1
845	Klrb1b	160	-0.250733	0.00138421	0.02976033	1
258	Calcoco1	160	-0.2507218	0.00138492	0.02976033	1
1319	Ring1	157	-0.253056	0.0013855	0.02976033	1
444	Ddx50	158	-0.252258	0.0013861	0.02976033	1
1581	Tmem109	145	-0.2630309	0.00139166	0.02985449	1
1231	Ppm1a	160	-0.2505962	0.00139289	0.02985559	1
1316	Rhox9	158	-0.2519948	0.00140276	0.02997825	1
172	Arl10	159	0.25121336	0.00140284	0.02997825	1
322	Cds2	160	-0.2504389	0.00140293	0.02997825	1
1501	Stat3	157	0.25277373	0.00140332	0.02997825	1
1491	Sptb	160	-0.2503997	0.00140544	0.02999835	1
1261	Ptchd1	160	-0.2503235	0.00141033	0.03007418	1
1466	Sntn	159	-0.2510658	0.00141228	0.03007418	1
560	Fam214b	158	-0.2518419	0.00141253	0.03007418	1
1103	Nudt2	155	-0.2541859	0.00141506	0.03009086	1
802	Itih3	156	0.25337428	0.00141568	0.03009086	1
430	Cysltr1	155	-0.254156	0.00141697	0.0300932	1
731	Helb	158	-0.2516897	0.00142231	0.03015085	1
134	Ank	159	-0.2508967	0.00142319	0.03015085	1
487	Dyx1c1	156	-0.2532561	0.00142324	0.03015085	1
383	Col3a1	158	-0.2514802	0.00143588	0.03036475	1
399	Cpe	155	-0.2538464	0.00143681	0.03036475	1
1543	Tbl2	160	0.2499139	0.00143691	0.03036475	1
822	Kcnj16	160	0.24987131	0.0014397	0.03037759	1
685	Gpr179	159	-0.2506397	0.00143991	0.03037759	1
454	Dhfr	156	0.25297515	0.00144136	0.03038315	1
656	Gm3579	156	-0.2528515	0.0014494	0.03052735	1
1737	Zfp692	159	-0.2504511	0.00145228	0.03055362	1
1663	Vamp3	159	-0.2504358	0.00145329	0.03055362	1

1225	Pomt2	156	0.25277732	0.00145425	0.03055362	1
610	Fzd9	160	-0.2495463	0.00146116	0.03067348	1
1725	Zdhhc4	154	0.2542605	0.00146239	0.03067412	1
420	Cul3	160	-0.2494765	0.0014658	0.03072032	1
1180	Peg3	159	-0.250184	0.00146998	0.03078252	1
240	Brd3	154	0.25407766	0.00147439	0.03080729	1
247	C2cd2	156	-0.252451	0.00147573	0.03080729	1
1153	Pank2	147	-0.2599156	0.00147666	0.03080729	1
943	Maged2	155	0.25321151	0.0014783	0.03080729	1
832	Kif12	156	0.25240109	0.00147905	0.03080729	1
1572	Tlcd2	160	-0.2492698	0.00147963	0.03080729	1
421	Cwc22	156	-0.2523642	0.0014815	0.03080729	1
809	Jarid2	156	0.25236366	0.00148154	0.03080729	1
819	Kcne2	160	-0.2492339	0.00148205	0.03080729	1
251	C87436	154	0.25392572	0.00148442	0.03083143	1
100	Adipor2	159	-0.249948	0.00148578	0.03083451	1
63	A830039N20Rik	160	-0.2491498	0.00148772	0.03084963	1
1609	Tpcn1	146	-0.2605051	0.00149499	0.03097508	1
1054	Nde1	160	-0.2489608	0.00150054	0.03106491	1
336	Cfap69	146	-0.2603573	0.00150463	0.03112423	1
566	Fam65b	160	-0.2488803	0.00150603	0.03112794	1
1245	Prelp	155	-0.2526518	0.00151577	0.03130388	1
1360	Samd11	145	-0.261035	0.0015179	0.03132227	1
242	Bspry	156	0.25178434	0.00152053	0.03135134	1
851	Krt71	158	-0.2501796	0.00152276	0.03136547	1
418	Cubn	156	-0.2517381	0.00152368	0.03136547	1
1133	Orai2	159	-0.2492583	0.00153286	0.03152881	1
1285	Rap1b	158	-0.2500149	0.0015341	0.03152893	1
630	Gas6	160	-0.2483889	0.00153994	0.03162347	1
859	Laptm4b	160	-0.2482941	0.00154657	0.03173396	1
326	Cenpa	155	0.25209759	0.00155374	0.03181802	1
1012	Mrrf	156	-0.251291	0.00155448	0.03181802	1
978	Mfsd7a	160	-0.2481791	0.00155463	0.03181802	1
1715	Zbp1	152	0.25449136	0.00155649	0.03181802	1
583	Fbxo7	160	-0.2481467	0.00155691	0.03181802	1
1067	Nfatc1	156	0.25123168	0.00155861	0.03182713	1
744	Hnrnph2	160	-0.2480718	0.0015622	0.03187487	1
11	1810030O07Rik	146	-0.2594534	0.00156487	0.03190388	1
1623	Tsx	156	0.25111227	0.00156695	0.03192065	1
450	Desi1	160	-0.2479594	0.00157016	0.03196045	1
365	Clk2	159	-0.2486924	0.00157249	0.03198229	1
1552	Tcirg1	160	0.2479069	0.00157389	0.03198517	1
834	Kif18a	155	0.25169521	0.00158184	0.0321212	1
1324	Ripply3	160	0.24773739	0.00158598	0.03217303	1
793	Irx2	160	-0.2477154	0.00158756	0.03217303	1

1704	Yif1b	151	-0.2548602	0.00158818	0.03217303	1
838	Klf8	158	-0.2491847	0.00159243	0.03222085	1
16	2310033P09Rik	158	0.24917576	0.00159307	0.03222085	1
199	Atp5c1	158	-0.2490606	0.00160133	0.03236206	1
1014	Msantd2	157	-0.249772	0.00160618	0.03243438	1
695	Grina	156	-0.2504725	0.00161233	0.03252858	1
297	Cd34	155	-0.2512447	0.00161385	0.03252858	1
902	LOC433602	157	-0.249654	0.00161468	0.03252858	1
1051	Ncald	160	-0.2472639	0.00162023	0.03261457	1
257	Calca	160	-0.2471148	0.00163115	0.03280846	1
1565	Tgfb1	160	-0.2469798	0.00164109	0.03295524	1
76	Acadl	160	-0.2469606	0.00164251	0.03295524	1
1011	Mrps5	143	0.26098062	0.00164263	0.03295524	1
438	Dcdc5	158	-0.2484788	0.00164362	0.03295524	1
571	Farsa	159	-0.247619	0.00165024	0.03306197	1
124	Aldoa	158	-0.2483672	0.00165184	0.03306805	1
854	Lag3	155	-0.2506892	0.00165414	0.03308795	1
827	Kctd3	138	-0.2653733	0.00165705	0.03312015	1
930	Ltf	155	0.2505538	0.0016641	0.03321378	1
229	Bglap3	153	0.25213882	0.0016656	0.03321378	1
1310	Rhbdd2	155	0.25053281	0.00166565	0.03321378	1
215	Bap1	159	-0.2473924	0.0016671	0.03321686	1
1265	Ptn	158	-0.2480887	0.00167254	0.03327359	1
547	Ezr	160	-0.2465575	0.00167257	0.03327359	1
673	Got2	159	-0.2472028	0.00168132	0.03342171	1
359	Cldn6	155	0.25028541	0.001684	0.03342894	1
1456	Sms	155	0.25028116	0.00168431	0.03342894	1
86	Acox3	138	-0.2649081	0.00168975	0.03351074	1
1223	Polr3k	160	-0.2463043	0.0016917	0.03352322	1
204	Atp6v0e	158	-0.2477744	0.00169619	0.0335407	1
10	1810024B03Rik	158	-0.247771	0.00169644	0.0335407	1
631	Gata2	160	-0.2462407	0.00169653	0.0335407	1
1575	Tm4sf1	159	-0.2469557	0.00170003	0.03355713	1
647	Glrb	160	-0.2461773	0.00170137	0.03355713	1
517	Emc2	158	-0.2477041	0.00170152	0.03355713	1
33	4833427G06Rik	157	-0.2484541	0.00170341	0.03355713	1
307	Cdh18	158	-0.247672	0.00170395	0.03355713	1
155	Aprt	156	0.24915802	0.00170935	0.03363749	1
1699	Wwp2	153	-0.2514951	0.00171344	0.03369187	1
1166	Pde3b	160	-0.245996	0.00171526	0.03370177	1
1230	Ppil6	158	-0.2474976	0.00171725	0.03371483	1
512	Elmo2	158	-0.2473861	0.00172581	0.03383861	1
750	Hpse2	159	-0.2466139	0.00172622	0.03383861	1
1346	Rps9	160	-0.2457917	0.00173104	0.03389908	1
1258	Psmc3	159	-0.2465395	0.00173196	0.03389908	1

903	LOC433610	159	-0.246477	0.0017368	0.03396774	1
664	Gm6878	158	-0.247186	0.00174126	0.03402696	1
1428	Slc35a1	160	-0.2456443	0.00174251	0.03402696	1
1264	Ptn	160	-0.2456152	0.00174478	0.03404323	1
642	Gin1	155	0.24946709	0.00174601	0.03404323	1
50	6330581N18Rik	159	-0.246285	0.00175175	0.03412905	1
884	LOC381076	160	0.24548332	0.00175511	0.03416055	1
308	Cdh22	159	-0.2462301	0.00175605	0.03416055	1
984	Mir99ahg	158	-0.2469498	0.00175966	0.03420464	1
813	Katnb1	147	-0.2558348	0.00176257	0.03423508	1
923	Lpar1	160	-0.2451649	0.00178028	0.03453434	1
1415	Slc20a2	159	-0.2459176	0.00178069	0.03453434	1
1022	Mtus2	160	-0.2450888	0.00178634	0.03461595	1
842	Klhl21	143	0.258995	0.00178762	0.03461595	1
941	Mafg	158	0.24651333	0.00179413	0.03468227	1
866	Lefty2	156	-0.2480571	0.00179467	0.03468227	1
972	Mepce	155	0.24882667	0.00179599	0.03468227	1
1002	Mptx1	160	0.24495049	0.00179742	0.03468227	1
961	Mbl2	156	0.2480169	0.00179786	0.03468227	1
5	1700036A12Rik	156	-0.2479171	0.00180579	0.03475963	1
333	Cfap126	158	-0.2463668	0.00180584	0.03475963	1
88	Acta1	160	-0.2448443	0.00180596	0.03475963	1
1576	Tm4sf1	160	-0.2447965	0.00180981	0.03478755	1
19	2310057J18Rik	159	0.24554926	0.00181014	0.03478755	1
1173	Pdia4	155	-0.2485987	0.00181409	0.03482318	1
355	Ckmt2	159	-0.2454724	0.00181634	0.03482318	1
608	Fxyd3	158	-0.2462206	0.00181759	0.03482318	1
1529	Syt7	160	-0.2446828	0.00181902	0.03482318	1
1159	Pcdhb16	160	-0.244671	0.00181998	0.03482318	1
442	Ddx19b	160	-0.2446682	0.0018202	0.03482318	1
1161	Pcdhgb6	153	-0.2500015	0.0018293	0.03497094	1
587	Fcgr2b	158	-0.2457744	0.00185388	0.03541434	1
543	Etl4	157	-0.2464313	0.00186312	0.03555036	1
26	2810007J24Rik	156	0.24719946	0.0018638	0.03555036	1
1492	Sqrdl	160	-0.244113	0.00186579	0.03556177	1
1357	S100b	160	-0.2440835	0.00186824	0.03556796	1
753	Hsd17b7	159	-0.2448243	0.0018694	0.03556796	1
105	Agpat3	160	-0.2440587	0.00187031	0.03556796	1
696	Grip2	160	-0.2439681	0.00187787	0.03566237	1
1007	Mrpl12	158	0.24548148	0.00187807	0.03566237	1
143	Ap3b1	156	-0.2469949	0.00188064	0.03568446	1
1129	Olfr984	159	-0.2445592	0.00189151	0.03586399	1
1142	Oxct1	158	-0.245251	0.0018973	0.03594714	1
423	Cxcl13	160	-0.2436817	0.00190192	0.0359825	1
1207	Plcxd2	160	-0.2436808	0.001902	0.0359825	1

1494	Srsf11	159	-0.2443879	0.00190592	0.03603	1
489	E2f1	160	-0.2435952	0.00190925	0.03606613	1
1448	Sln	160	-0.2435756	0.00191091	0.03607064	1
1650	Uhrf1	151	0.25052834	0.00191832	0.0361838	1
1487	Spg21	160	-0.2434467	0.00192188	0.03620863	1
1656	Usp19	160	-0.2434396	0.00192248	0.03620863	1
1281	Rala	156	0.24641612	0.00192905	0.03629682	1
1616	Trim39	155	0.24718607	0.00193001	0.03629682	1
638	Gfra2	158	-0.2448043	0.00193508	0.03635122	1
377	Cntn4	158	-0.2447964	0.00193576	0.03635122	1
1518	Sybu	158	-0.2447618	0.00193872	0.03637995	1
872	Lhfpl2	158	-0.2447376	0.00194079	0.03639192	1
405	Crb1	159	-0.243871	0.00195002	0.03653808	1
1707	Yjefn3	158	-0.244576	0.00195466	0.03659814	1
1570	Timm50	155	0.24685426	0.0019582	0.03662462	1
192	Atg4b	159	-0.2437457	0.00196084	0.03662462	1
114	Akna	158	-0.2444961	0.00196155	0.03662462	1
585	Fbxo9	160	-0.2429827	0.00196183	0.03662462	1
1300	Rest	159	-0.243705	0.00196437	0.03664525	1
963	Mcat	159	-0.2436574	0.0019685	0.03668191	1
1149	Pafah1b1	160	-0.2428979	0.00196922	0.03668191	1
567	Fam73b	157	-0.245127	0.0019732	0.03671258	1
1280	Rag2	157	-0.2451207	0.00197375	0.03671258	1
44	4933435E02Rik	157	-0.2450992	0.00197561	0.03672034	1
393	Cox6c	158	-0.2442945	0.00197904	0.03674793	1
1677	Vti1a	160	-0.2427748	0.00197998	0.03674793	1
701	Gsc	152	-0.2488747	0.00198946	0.03689697	1
954	Marcksl1	16	-0.7115974	0.00199113	0.03690112	1
556	Fam178b	154	0.24722022	0.00199448	0.03693629	1
678	Gpr133	160	-0.2425848	0.00199669	0.03695043	1
1400	Sin3a	159	-0.2433163	0.00199836	0.03695451	1
1013	Mrs2	158	-0.2440227	0.00200285	0.03701051	1
495	Ecel1	134	0.26457866	0.00200648	0.0370441	1
431	D10Jhu81e	156	0.24550467	0.00200757	0.0370441	1
1250	Prlr	154	0.24704043	0.00201012	0.03705329	1
506	Eif3j1	157	-0.2446946	0.00201098	0.03705329	1
1486	Speer1-ps1	159	-0.2431491	0.00201315	0.03706643	1
1612	Trap1a	154	0.24698279	0.00201516	0.03707658	1
1031	Mx2	160	0.24231492	0.00202066	0.03715098	1
1457	Smu1	160	-0.2422546	0.00202605	0.03716627	1
540	Esyt3	160	0.24224614	0.00202681	0.03716627	1
471	Dnajc4	158	-0.2437481	0.00202716	0.03716627	1
125	Alg1	136	-0.2624079	0.00202733	0.03716627	1
1398	Siah1b	156	-0.2451517	0.00203876	0.03731007	1
671	Gon4l	158	-0.2436159	0.00203896	0.03731007	1

648	Gm10010	160	0.24210403	0.00203957	0.03731007	1
20	2610002J02Rik	159	-0.2428333	0.00204135	0.03731578	1
295	Cct8	160	-0.2420616	0.0020434	0.03732646	1
36	4922502D21Rik	160	0.24203348	0.00204593	0.03734595	1
1447	Slco2a1	159	-0.2426996	0.00205339	0.03742946	1
1605	Tnni3	158	-0.2434445	0.00205435	0.03742946	1
940	Maea	160	-0.2419342	0.00205492	0.03742946	1
598	Foxb1	160	-0.2418882	0.00205909	0.03747693	1
1355	Rxfp3	156	0.24490886	0.00206047	0.03747693	1
982	Mipep	156	-0.2448547	0.00206534	0.03751449	1
1276	Rab34	158	-0.2433214	0.00206548	0.03751449	1
428	Cyp4v3	159	-0.2425389	0.00206796	0.03753279	1
37	4930430A15Rik	156	-0.2447877	0.00207137	0.03756797	1
1100	Nudc	160	-0.2416951	0.00207669	0.0376318	1
1016	Msmo1	160	-0.2416768	0.00207837	0.0376318	1
1511	Sugp1	133	-0.2646747	0.00207933	0.0376318	1
1509	Suco	158	-0.2431068	0.00208499	0.03770755	1
81	Ace	160	-0.2414857	0.00209594	0.03782903	1
1093	Nt5c2	160	-0.2414622	0.00209812	0.03782903	1
1618	Trmt112	158	-0.2429611	0.00209834	0.03782903	1
1558	Tecta	160	-0.2414149	0.00210249	0.03782903	1
193	Atg5	156	-0.2444333	0.00210359	0.03782903	1
223	Bcl10	157	-0.2436588	0.00210416	0.03782903	1
244	Btn1a1	152	0.24756375	0.00210511	0.03782903	1
1353	Runx1	158	-0.2428833	0.00210549	0.03782903	1
955	Marco	156	0.24441077	0.00210565	0.03782903	1
595	Fmn2	159	-0.2421161	0.00210672	0.03782903	1
1705	Yipf1	160	-0.2413549	0.00210805	0.03782903	1
705	Gsto1	160	-0.2413149	0.00211176	0.03786502	1
734	Hhip	160	-0.2413013	0.00211303	0.03786502	1
278	Ccdc177	156	-0.2442738	0.00211823	0.03793151	1
1416	Slc22a12	155	0.24499123	0.00212353	0.03799978	1
620	Gad2	158	0.24265061	0.00212703	0.03803566	1
505	Eif3g	158	-0.2426205	0.00212983	0.03804929	1
1386	Sfrp2	158	0.24261032	0.00213078	0.03804929	1
880	Lmo4	160	-0.2410536	0.00213617	0.03811871	1
1752	Zpr1	156	0.24397321	0.00214609	0.03826886	1
284	Ccdc97	159	-0.2416686	0.00214847	0.03828452	1
56	9130204K15Rik	160	-0.2409057	0.0021501	0.0382868	1
183	Asb4	160	0.24088448	0.00215211	0.03829578	1
372	Cndp2	159	0.24159575	0.00215534	0.03831003	1
291	Ccrl2	156	0.24386795	0.00215592	0.03831003	1
1425	Slc28a2	156	0.24381384	0.00216099	0.03837335	1
1274	R74862	159	-0.2414225	0.00217175	0.0385321	1
1414	Slc20a1	160	-0.240665	0.00217295	0.0385321	1

549	F3	152	0.24679031	0.00217618	0.0385624	1
1449	Smc3	159	-0.241295	0.0021839	0.03865563	1
301	Cdadc1	159	-0.241289	0.00218447	0.03865563	1
697	Grm4	160	-0.2404978	0.00218895	0.03870798	1
799	Itfg3	160	-0.2404723	0.0021914	0.0387244	1
989	Mmp14	160	-0.2403875	0.00219957	0.03884016	1
570	Fars2	155	0.24416323	0.002201	0.03884016	1
1443	Slc6a8	137	-0.2594418	0.00220258	0.03884112	1
157	Aqp4	160	-0.2403189	0.00220619	0.03887777	1
553	Fam131b	158	-0.2417963	0.00220785	0.03888007	1
1649	Ugt2a2	158	-0.2417713	0.00221025	0.03889552	1
1690	Wfikkn2	158	-0.2417035	0.0022168	0.03898379	1
110	Aifm3	160	-0.2401749	0.00222015	0.03901424	1
187	Aspg	160	-0.2401601	0.00222159	0.03901424	1
1345	Rps6ka6	152	0.24623855	0.0022282	0.03910324	1
200	Atp5e	160	-0.2399851	0.00223868	0.03925529	1
254	Cab39l	160	-0.2399722	0.00223994	0.03925529	1
480	Drg2	158	-0.2414282	0.00224352	0.03929088	1
853	L3mbtl4	160	-0.2399162	0.00224545	0.03929765	1
1716	Zbtb11	159	-0.2406309	0.00224819	0.0393186	1
1463	Sncb	158	-0.2413426	0.00225189	0.03935625	1
1070	Ngfr	160	0.23979628	0.00225725	0.03942299	1
1472	Sod3	158	0.24120662	0.00226525	0.03953544	1
349	Chrnd	160	0.23968747	0.00226802	0.03954121	1
1561	Tex11	159	-0.2404228	0.00226868	0.03954121	1
890	LOC432635	157	-0.2418579	0.00227556	0.03963121	1
775	Igfbp5	159	-0.2403268	0.00227819	0.03963121	1
1613	Treml1	156	-0.2425907	0.00227851	0.03963121	1
42	4933400C23Rik	158	-0.2409324	0.0022924	0.03984548	1
651	Gm11538	158	0.24089024	0.00229659	0.03989119	1
516	Emb	160	-0.2393631	0.00230039	0.03992983	1
422	Cwf19l2	158	-0.2408213	0.00230347	0.03995616	1
1666	Vcam1	160	-0.2392967	0.00230707	0.03999134	1
1202	Pkia	142	-0.2537466	0.00231103	0.04003271	1
1661	Uts2b	160	-0.2392319	0.0023136	0.04004996	1
1515	Suv39h1	156	-0.2422061	0.00231666	0.04007568	1
1644	Ublcp1	160	-0.2391605	0.00232082	0.0401007	1
1603	Tnfsf14	155	0.24292891	0.00232125	0.0401007	1
1527	Syt2	160	-0.2391018	0.00232677	0.04015638	1
582	Fbxo2	158	0.24054153	0.0023316	0.04015638	1
629	Gar1	159	-0.2397659	0.0023345	0.04015638	1
1598	Tmem64	160	-0.2390195	0.00233514	0.04015638	1
1068	Nfil3	152	0.24513055	0.00233608	0.04015638	1
843	Klk14	156	-0.242011	0.00233622	0.04015638	1
214	Baiap3	160	0.23899252	0.00233789	0.04015638	1

710	H2-Ab1	159	0.23971394	0.00233978	0.04015638	1
78	Acat3	156	-0.24196	0.00234136	0.04015638	1
938	Macrod1	155	0.24272462	0.00234172	0.04015638	1
1451	Smchd1	158	-0.2404406	0.00234182	0.04015638	1
329	Cenpq	149	0.24744798	0.00234614	0.04020329	1
508	Eif4a3	160	0.23889518	0.00234783	0.04020516	1
1698	Wwc1	160	-0.2387627	0.00236141	0.04041063	1
346	Chrna2	160	0.23872353	0.00236545	0.04045253	1
347	Chrna3	159	-0.2394374	0.00236805	0.04046987	1
91	Acvrl1	160	-0.2386249	0.00237563	0.04057162	1
905	LOC433701	158	-0.2400945	0.00237719	0.04057162	1
1735	Zfp609	157	-0.240782	0.00238389	0.04065871	1
412	Csnk1g3	156	-0.2414672	0.00239156	0.0407434	1
1539	Tas2r134	158	0.23995038	0.00239206	0.0407434	1
864	Lef1	61	0.38175679	0.00239889	0.04083102	1
1528	Syt3	160	-0.2383864	0.00240041	0.04083102	1
1578	Tmem101	158	0.23984164	0.00240333	0.04085346	1
252	C87499	154	0.24285829	0.0024069	0.04088687	1
38	4930463O16Rik	159	-0.2390379	0.00240946	0.04090292	1
1299	Reep6	152	0.24433121	0.00241682	0.04096701	1
667	Gm973	158	-0.2396909	0.00241904	0.04096701	1
1724	Zcchc9	152	0.24430922	0.00241908	0.04096701	1
331	Cep41	154	0.24273447	0.00241967	0.04096701	1
233	Blzf1	159	-0.2389082	0.00242304	0.04099683	1
1646	Uck1	160	-0.2381536	0.00242483	0.04099987	1
847	Kpna2	160	-0.2381084	0.00242959	0.04102977	1
1162	Pcm1	160	-0.2381062	0.00242982	0.04102977	1
523	Enpp2	160	-0.2380733	0.0024333	0.0410613	1
390	Cops3	159	-0.2387112	0.0024438	0.04121113	1
1306	Rgma	160	-0.2379529	0.00244605	0.04122186	1
451	Desi2	160	-0.2379197	0.00244958	0.04125403	1
15	2310030G06Rik	156	0.24088987	0.00245159	0.04126064	1
1026	Mup4	156	0.2408483	0.00245597	0.04130698	1
737	Hipk2	158	-0.2393091	0.00245924	0.04133479	1
1279	Rad51	155	-0.241456	0.00247253	0.04153066	1
150	Apobec3	158	0.23911676	0.00247973	0.04162411	1
1333	Rnf217	159	-0.2383319	0.00248423	0.04167219	1
32	4833403I15Rik	152	-0.2436649	0.00248606	0.04167544	1
1433	Slc39a7	159	-0.2382886	0.00248888	0.04169524	1
1085	Npepl1	158	-0.2390066	0.00249152	0.04171216	1
482	Dstn	160	-0.2375063	0.0024939	0.04171965	1
416	Ctnnb1	160	-0.2374772	0.00249704	0.04171965	1
1330	Rnf121	153	0.2427692	0.00249762	0.04171965	1
481	Dsg4	160	-0.2374531	0.00249965	0.04171965	1
1596	Tmem25	160	-0.237441	0.00250097	0.04171965	1

1437	Slc4a1	155	0.24118044	0.0025018	0.04171965	1
158	Aqp4	156	-0.2403992	0.00250369	0.04172384	1
1541	Tbc1d16	159	0.2381321	0.00250576	0.04173102	1
1622	Tspyl4	160	-0.2372582	0.00252084	0.04195472	1
302	Cdan1	159	-0.2379559	0.0025249	0.04199477	1
663	Gm597	159	-0.2378484	0.00253664	0.04214842	1
437	Dcbld1	155	0.24084883	0.00253745	0.04214842	1
84	Acot11	158	-0.2385467	0.00254134	0.04217823	1
1697	Wscd1	158	-0.2385356	0.00254255	0.04217823	1
374	Cnnm2	156	-0.2399917	0.00254771	0.04223629	1
1208	Pld2	156	0.23992142	0.00255538	0.04232319	1
1522	Synj2bp	160	-0.2369355	0.00255628	0.04232319	1
1460	Snap25	159	-0.2376071	0.00256317	0.04240973	1
795	Irx4	160	-0.236846	0.00256618	0.042432	1
1150	Pafah2	155	0.24055083	0.00256987	0.04246538	1
327	Cenpf	155	-0.2405328	0.00257184	0.04247042	1
1052	Ncald	160	-0.2367058	0.00258177	0.0426068	1
1383	Serpinf1	156	-0.2396335	0.00258699	0.04266526	1
1292	Rbmy	158	-0.2381149	0.00258893	0.04266965	1
1156	Parvg	152	0.24268841	0.00259076	0.04267219	1
29	3300005D01Rik	156	-0.2395815	0.00259274	0.04267713	1
1171	Pdgfrb	156	-0.2395422	0.00259709	0.04272115	1
904	LOC433666	158	-0.2379985	0.0026019	0.04277257	1
1551	Tcf21	158	0.23791557	0.00261118	0.0428974	1
742	Hnrnpa3	159	-0.2371471	0.00261444	0.0429234	1
654	Gm205	159	-0.2371116	0.00261844	0.04293193	1
179	Arrdc3	160	-0.2363539	0.00262129	0.04293193	1
981	Miat	146	-0.2472557	0.00262297	0.04293193	1
1062	Nek11	158	-0.237809	0.00262314	0.04293193	1
1060	Nedd9	160	-0.2363274	0.00262429	0.04293193	1
1268	Ptpn21	158	-0.2377917	0.00262508	0.04293193	1
962	Mc4r	158	0.23768988	0.00263657	0.04309212	1
353	Ciita	158	-0.2376733	0.00263845	0.04309518	1
1092	Nrxn1	160	-0.2361817	0.00264083	0.04309618	1
1128	Olfr705	159	-0.2368912	0.00264336	0.04309618	1
277	Ccdc171	160	-0.2361513	0.00264429	0.04309618	1
264	Carhsp1	155	0.23987071	0.00264528	0.04309618	1
156	Aqp2	160	-0.2360783	0.00265262	0.04318819	1
1482	Spata16	159	0.23677706	0.00265636	0.04322137	1
31	4732414G09Rik	156	0.23893877	0.00266474	0.04333014	1
1312	Rhno1	160	0.23578815	0.00268597	0.04363514	1
1082	Nod2	160	-0.235768	0.0026883	0.04363514	1
310	Cdh24	160	-0.235765	0.00268864	0.04363514	1
605	Frs3	160	-0.2357059	0.00269548	0.04365558	1
1635	Tymp	154	-0.2401804	0.00269694	0.04365558	1

509	Eif4e2	158	-0.2371553	0.0026976	0.04365558	1
588	Fezf1	160	0.23567689	0.00269885	0.04365558	1
968	Med19	158	-0.2371421	0.00269913	0.04365558	1
973	Mest	152	0.24168744	0.00270222	0.04365558	1
378	Cntnap3	159	-0.2363702	0.00270315	0.04365558	1
1481	Spag8	159	-0.2363662	0.00270362	0.04365558	1
350	Chst14	160	-0.2355768	0.0027105	0.0437232	1
861	Lcat	160	-0.2355704	0.00271124	0.0437232	1
1548	Tceal8	158	-0.2369909	0.00271663	0.04378245	1
1119	Olfr1357	159	0.23621556	0.00272112	0.04382714	1
282	Ccdc8	159	0.2361455	0.00272931	0.04393113	1
514	Elovl3	156	0.23822584	0.00274672	0.04416335	1
275	Ccdc115	155	0.23897878	0.0027472	0.04416335	1
1632	Tulp3	158	-0.2367126	0.00274911	0.04416607	1
1291	Rbmx2	155	0.23894231	0.00275144	0.04417576	1
249	C5ar1	157	-0.2373274	0.00276413	0.04434861	1
1419	Slc25a18	160	-0.2351075	0.00276569	0.04434861	1
1379	Serpinb10	155	0.23880429	0.00276755	0.04435051	1
524	Enpp5	156	-0.2379948	0.00277378	0.04442225	1
174	Arl16	160	-0.2350248	0.00277552	0.04442225	1
360	Clec18a	160	-0.2349684	0.00278224	0.04450193	1
394	Cox6c	160	-0.2348818	0.00279259	0.04462266	1
1627	Ttll5	134	-0.2563354	0.0027933	0.04462266	1
1544	Tbl3	160	-0.2347759	0.0028053	0.04477861	1
965	Mcm4	155	0.23847291	0.00280658	0.04477861	1
748	Hoxa9	10	-0.8322527	0.00281442	0.04485483	1
1732	Zfp354b	159	-0.2354238	0.00281488	0.04485483	1
142	Ap1b1	160	-0.2346348	0.0028223	0.04494503	1
625	Galk1	154	0.23906512	0.0028268	0.04498853	1
436	Dbx1	156	-0.2375153	0.00283068	0.04502214	1
1185	Pex19	140	-0.250404	0.00284557	0.04523066	1
1728	Zfp180	158	-0.2358767	0.0028488	0.04524894	1
860	Larp1b	160	-0.2343929	0.00285168	0.04524894	1
1262	Ptgfrn	138	-0.2521286	0.00285205	0.04524894	1
472	Dnali1	158	-0.2357972	0.00285846	0.04532237	1
459	Dlk1	160	0.23427202	0.00286647	0.04541193	1
613	Gaa	160	0.23426221	0.00286767	0.04541193	1
1504	Stmn4	160	-0.2342314	0.00287145	0.04544352	1
171	Arid5b	158	-0.2356646	0.00287461	0.04546521	1
1619	Tsc2	160	-0.2341763	0.00287822	0.04549414	1
341	Chchd3	160	-0.2341385	0.00288287	0.04553938	1
1573	Tle3	155	-0.2378133	0.00288574	0.04555644	1
593	Fgf13	160	-0.2340754	0.00289066	0.04556108	1
823	Kcnj5	160	-0.2340507	0.00289372	0.04556108	1
1684	Wac	158	-0.2355035	0.00289435	0.04556108	1

1040	N4bp2l1	147	0.2440378	0.00289462	0.04556108	1
950	Map3k4	158	-0.2354984	0.00289498	0.04556108	1
796	Isca2	160	-0.2340241	0.00289701	0.04556487	1
1037	Myrfl	159	-0.2347085	0.00290209	0.04561649	1
48	5730455P16Rik	158	-0.2354056	0.00290642	0.04565117	1
752	Hsd17b2	156	0.23687868	0.00290788	0.04565117	1
771	Ifnar1	160	-0.233914	0.00291067	0.04566052	1
589	Fezf1	160	0.23390276	0.00291206	0.04566052	1
1206	Plcl1	159	-0.2345734	0.00291883	0.04573859	1
1536	Taok3	158	0.23525567	0.00292497	0.04580658	1
1571	Tktl2	157	-0.2358831	0.00293875	0.04594459	1
655	Gm2a	160	-0.2336866	0.00293906	0.04594459	1
924	Lrp1	160	-0.2336854	0.0029392	0.04594459	1
931	Ltn1	160	-0.2335597	0.002955	0.04616322	1
768	Idh1	158	0.23492853	0.00296583	0.04629561	1
812	Jund	160	-0.2334638	0.00296711	0.04629561	1
1640	Ube2b	160	-0.2333455	0.0029821	0.0464545	1
688	Gprc5b	160	-0.2333364	0.00298326	0.0464545	1
967	Med1	159	-0.2340553	0.00298385	0.0464545	1
410	Cryba2	159	-0.2340494	0.00298459	0.0464545	1
766	Id1	158	-0.2347421	0.00298934	0.0464814	1
1708	Ythdf2	154	0.237727	0.00299005	0.0464814	1
25	2700081O15Rik	160	-0.2332667	0.00299212	0.0464814	1
1526	Syt11	160	-0.233255	0.00299362	0.0464814	1
719	Hax1	160	-0.2331411	0.00300817	0.0466789	1
348	Chrna3	160	-0.2331196	0.00301093	0.04669329	1
1672	Vmn1r66	159	-0.2337958	0.0030169	0.0467168	1
537	Erlin2	155	0.2367526	0.00301728	0.0467168	1
417	Ctps	145	-0.2446664	0.00301812	0.0467168	1
112	Aire	160	0.23305057	0.00301979	0.0467168	1
443	Ddx42	156	-0.235961	0.0030225	0.04672541	1
260	Cald1	158	0.23445092	0.00302641	0.04672541	1
817	Kcnab2	160	-0.2329799	0.00302889	0.04672541	1
1136	Ormdl3	156	0.23590682	0.00302939	0.04672541	1
1099	Nubp1	160	0.23297495	0.00302952	0.04672541	1
519	Emilin2	156	0.23585016	0.00303662	0.04678698	1
1611	Tprkb	159	-0.2336378	0.00303719	0.04678698	1
920	LOC546168	157	-0.2350688	0.00304154	0.04682191	1
733	Hhip	152	-0.2388422	0.00304313	0.04682191	1
184	Asb8	160	-0.2328084	0.00305106	0.04691553	1
1077	Nisch	160	-0.232754	0.00305811	0.04699562	1
561	Fam221b	157	0.23491728	0.00306102	0.04701201	1
1549	Tcerg1	160	-0.2327071	0.00306421	0.04703256	1
463	Dmd	159	-0.2333975	0.00306828	0.04706667	1
1151	Pald1	160	-0.2326565	0.0030708	0.04707704	1

1323	Ripk4	157	-0.2347922	0.00307719	0.04711857	1
1628	Ttll6	158	-0.234057	0.00307721	0.04711857	1
1239	Ppp3r1	159	-0.2332569	0.00308661	0.04720753	1
534	Epm2aip1	157	0.23471866	0.00308673	0.04720753	1
119	Aldh18a1	152	0.23841509	0.00309753	0.04730755	1
401	Cpt2	157	-0.2346197	0.00309961	0.04730755	1
1713	Ywhag	138	-0.2500218	0.00310149	0.04730755	1
194	Atg9b	156	-0.2353447	0.00310176	0.04730755	1
635	Gbp2	156	-0.2353361	0.00310287	0.04730755	1
1476	Sorcs2	160	-0.2324001	0.00310442	0.04730755	1
788	Iqch	158	-0.2337961	0.00311128	0.04737656	1
1461	Snap91	160	-0.232336	0.00311288	0.04737656	1
787	Iqcg	158	-0.2337714	0.00311453	0.04737656	1
241	Brs3	158	0.23375643	0.0031165	0.04737818	1
64	A930012M21Rik	158	-0.2337176	0.0031216	0.04742743	1
913	LOC436099	160	-0.2322484	0.00312447	0.0474427	1
806	Izumo3	159	-0.232933	0.0031292	0.04748626	1
844	Klk1b8	155	0.23581454	0.00313809	0.04753102	1
1668	Vdac2	160	-0.23213	0.0031402	0.04753102	1
1309	Rgs9	158	0.23357419	0.00314053	0.04753102	1
1313	Rhobtb2	158	-0.2335665	0.00314156	0.04753102	1
1234	Ppp1r13b	159	-0.2328391	0.00314164	0.04753102	1
1296	Rdh10	156	-0.2350241	0.00314372	0.04753102	1
126	Alg9	155	0.23576023	0.00314522	0.04753102	1
145	Apba2	160	-0.2320007	0.00315745	0.04766468	1
1633	Txndc11	156	0.23490695	0.00315918	0.04766468	1
328	Cenpm	156	0.23490321	0.00315968	0.04766468	1
424	Cyc1	160	-0.2319481	0.00316449	0.04769989	1
550	F8a	156	-0.2348573	0.00316576	0.04769989	1
893	LOC432742	159	-0.2325788	0.00317637	0.04783152	1
1169	Pdgfra	159	-0.2325365	0.00318205	0.0478887	1
1655	Usp18	158	-0.2332261	0.00318691	0.04793348	1
1445	Slc7a6	159	0.23237233	0.00320418	0.04814294	1
1221	Pofut2	160	-0.2316505	0.00320461	0.04814294	1
545	Exosc9	159	0.23230445	0.00321336	0.04823614	1
340	Chaf1a	160	0.23156494	0.00321623	0.04823614	1
1008	Mrpl23	159	-0.2322758	0.00321724	0.04823614	1
323	Ceacam3	160	0.23154672	0.00321871	0.04823614	1
1681	Vwa3a	159	-0.2322534	0.00322029	0.04823614	1
8	1700123I01Rik	158	0.23292166	0.00322798	0.04830686	1
868	Lepr	158	-0.2329156	0.0032288	0.04830686	1
515	Elp2	156	-0.2343208	0.00323757	0.04838482	1
1113	Oat	160	-0.2314035	0.00323825	0.04838482	1
47	5530401A14Rik	159	-0.2321107	0.00323972	0.04838482	1
1745	Zfpm2	160	-0.2313336	0.00324784	0.04847772	1

1121	Olfr1494	159	-0.232006	0.00325403	0.04854173	1
644	Gjb2	160	-0.2312266	0.00326255	0.04863642	1
1362	Samhd1	158	0.232656	0.0032642	0.04863642	1
907	LOC433761	152	0.23711326	0.0032688	0.04864896	1
94	Adamts19	160	0.23118082	0.00326886	0.04864896	1
1235	Ppp1r15a	154	-0.2354773	0.00328378	0.04881338	1
337	Cfh	157	-0.2332308	0.00328559	0.04881338	1
1256	Psenen	157	-0.2332249	0.00328641	0.04881338	1
1394	Sh3gl2	160	-0.2310456	0.00328758	0.04881338	1
743	Hnrnpf	156	-0.2339346	0.00329018	0.04882356	1
619	Gad1	158	0.23243876	0.0032941	0.04884924	1
639	Gfral	159	-0.2317034	0.00329575	0.04884924	1
13	2310002L09Rik	156	0.23387744	0.00329804	0.04885472	1
993	Mmp8	159	-0.2316338	0.00330541	0.04891516	1
645	Gjd3	160	-0.2309109	0.00330632	0.04891516	1
216	BC048671	156	0.23380596	0.00330788	0.04891516	1
818	Kcnd2	158	-0.2322765	0.00331659	0.04896143	1
698	Grm8	158	-0.2322664	0.00331799	0.04896143	1
1314	Rhot1	160	-0.230824	0.00331845	0.04896143	1
452	Dgcr8	157	-0.232991	0.0033187	0.04896143	1
1630	Ttyh3	158	-0.2321392	0.00333572	0.04918401	1
1029	Mut	157	-0.2328475	0.00333866	0.04919882	1
939	Mad2l1	158	-0.2320836	0.0033435	0.04922386	1
800	Itga10	158	0.23207847	0.00334422	0.04922386	1
1667	Vcpkmt	159	0.23132967	0.00334794	0.0492501	1
500	Efhc1	158	-0.2320308	0.0033509	0.04926522	1
67	Aagab	159	-0.2312268	0.00336243	0.0494052	1
289	Ccni	146	-0.2411471	0.0033663	0.0494052	1
1485	Spdef	145	-0.2419663	0.00336633	0.0494052	1
368	Cluap1	157	-0.2326366	0.00336818	0.0494052	1
236	Bms1	157	-0.2326033	0.00337287	0.04944541	1
397	Cpa2	158	0.23178675	0.00338531	0.04955697	1
1513	Sun2	160	0.23034544	0.00338602	0.04955697	1
781	Il1rl2	160	0.2303434	0.00338631	0.04955697	1
1058	Necab1	160	-0.2302733	0.00339632	0.04967483	1
1106	Nwd2	160	-0.2302505	0.00339957	0.04968991	1
693	Grin2d	160	-0.2302388	0.00340125	0.04968991	1
1199	Pip4k2c	160	0.23016096	0.0034124	0.04981638	1
562	Fam222b	138	-0.2475725	0.0034161	0.04981638	1
1458	Smyd2	158	-0.231568	0.00341642	0.04981638	1
386	Col9a1	152	0.23603022	0.00341774	0.04981638	1
217	BC049702	158	-0.2315226	0.00342291	0.04986324	1
600	Foxk1	158	-0.2314896	0.00342764	0.04988292	1
1015	Msi2	160	-0.2300512	0.00342818	0.04988292	1
1381	Serpinb1b	160	-0.2300004	0.00343552	0.04992922	1

97	Adcy5	157	0.23215687	0.00343623	0.04992922	1
1747	Zfyve19	158	0.23141321	0.00343859	0.04992922	1
951	Map3k6	157	-0.2321361	0.0034392	0.04992922	1
1251	Prm1	158	-0.2313642	0.00344563	0.049994	1
